# Polarization multiplexed diffractive computing: all-optical implementation of a group of linear transformations through a polarization-encoded diffractive network

**DOI:** 10.1038/s41377-022-00849-x

**Published:** 2022-05-26

**Authors:** Jingxi Li, Yi-Chun Hung, Onur Kulce, Deniz Mengu, Aydogan Ozcan

**Affiliations:** 1 Electrical and Computer Engineering Department, University of California, Los Angeles, CA 90095 USA; 2 Bioengineering Department, University of California, Los Angeles, CA 90095 USA; 3 California NanoSystems Institute (CNSI), University of California, Los Angeles, CA 90095 USA

**Keywords:** Optical techniques, Applied optics, Transformation optics

## Abstract

Research on optical computing has recently attracted significant attention due to the transformative advances in machine learning. Among different approaches, diffractive optical networks composed of spatially-engineered transmissive surfaces have been demonstrated for all-optical statistical inference and performing arbitrary linear transformations using passive, free-space optical layers. Here, we introduce a polarization-multiplexed diffractive processor to all-optically perform multiple, arbitrarily-selected linear transformations through a single diffractive network trained using deep learning. In this framework, an array of pre-selected linear polarizers is positioned between trainable transmissive diffractive materials that are isotropic, and different target linear transformations (complex-valued) are uniquely assigned to different combinations of input/output polarization states. The transmission layers of this polarization-multiplexed diffractive network are trained and optimized via deep learning and error-backpropagation by using thousands of examples of the input/output fields corresponding to each one of the complex-valued linear transformations assigned to different input/output polarization combinations. Our results and analysis reveal that a single diffractive network can successfully approximate and all-optically implement a group of arbitrarily-selected target transformations with a negligible error when the number of trainable diffractive features/neurons (*N*) approaches $N_pN_iN_o$, where *N*_*i*_ and *N*_*o*_ represent the number of pixels at the input and output fields-of-view, respectively, and *N*_*p*_ refers to the number of unique linear transformations assigned to different input/output polarization combinations. This polarization-multiplexed all-optical diffractive processor can find various applications in optical computing and polarization-based machine vision tasks.

## Abstract (web summary)

All-optical computation of a group of linear transforms is achieved using a polarization-encoded diffractive optical processor.

## Introduction

With the increasing global demand for machine learning and computing in general, using light to perform computation has been a rapidly growing focus area of optics and photonics^1–5^. The research on optical computing has a long history spanning decades of exciting research and development efforts^6–31^. Motivated by the massive success of artificial intelligence and deep learning, in specific, a myriad of new hardware designs for optical computing have been reported recently, including, e.g., on-chip integrated photonic circuits^16–22^, free-space optical platforms^23–28^, and others^29–31^. Among these different optical computing systems, the integration of successive transmissive diffractive layers (forming an optical network) has been demonstrated for optical information processing, such as object classification^23,32–43^, image reconstruction^38,44^, all-optical phase recovery and quantitative phase imaging^45^, and logic operations^46–48^. A diffractive network is trained using deep learning and error-backpropagation methods implemented in a digital computer, after which the resulting transmissive layers are fabricated to form a physical network that computes based on the diffraction of the input light through these spatially-engineered transmissive layers. Because the computational task is completed as the light passes through thin and passive optical elements, this approach is very fast, and the inference process does not consume power except for the illumination light. It is also scalable since an increase in the input field-of-view (FOV) can be handled by fabricating larger transmissive layers and/or deeper diffractive designs with more successive layers positioned one after another. Furthermore, both the phase and the amplitude information channels of the input scene/FOV can be processed by a diffractive optical network, without the need for phase retrieval or digitizing, vectorizing an image of the scene, which makes diffractive computing highly desirable for machine vision applications^38,44^. Harnessing light-matter interactions using engineered diffractive surfaces also enabled the inverse design of optical elements for e.g., spatially-controlled wavelength demultiplexing^49^, pulse engineering^50^, and orbital angular momentum multiplexing/demultiplexing^51,52^. It has also been shown that a diffractive network can be trained by optimizing its diffractive layers to perform an arbitrary complex-valued linear transformation between its input and output fields-of-view, demonstrating its computing capability for complex-valued matrix-vector operations at the speed of light propagation through a passive diffractive system.

All these results highlight the unique capabilities of diffractive networks to manipulate various physical properties of light, including e.g., its amplitude and phase distribution, spatial frequency, spectral bandwidth, orbital angular momentum, for performing specific computational tasks that are desired. As another important physical property of light, polarization specifies the geometrical orientation of electromagnetic wave oscillations. Utilizing the polarization state of light has played a pivotal role in numerous applications, including telecommunications^53–55^, imaging^56–61^, sensing^62–64^, computing^65^, and displays^66,67^. For example, polarization-division multiplexing (PDM) has been used in telecommunication systems to permit two channels of information to be simultaneously transmitted using orthogonal polarization states over a single wavelength^54,68^.

**Fig. 1:**
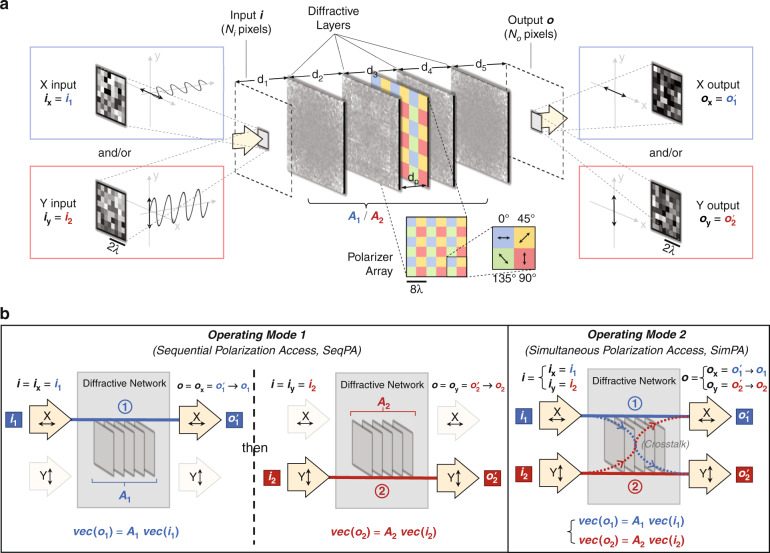
Schematic of polarization-multiplexed all-optical diffractive computing. **a** Optical layout of the polarization-encoded diffractive network, where four isotropic diffractive layers and one array of linear polarizers are jointly used to perform two distinct, complex-valued linear transformations between the input field ***i*** and the output field ***o*** by using polarization encoding/decoding at the input/output FOVs. **b** Schematic for the sequential polarization access (SeqPA, left) mode and the simultaneous polarization access (SimPA, right) mode that can be used to operate the 2-channel polarization-multiplexed diffractive network

Here, we report the design of polarization-multiplexed diffractive optical networks to perform a group of arbitrary linear transformations using a common set of diffractive layers that are jointly optimized to all-optically perform each one of the target complex-valued linear transformations at a different combination of input/output polarization states. In our earlier work^69^, we showed that a diffractive optical network composed of spatially-engineered layers could all-optically perform an arbitrary complex-valued linear transformation between an input and output field-of-view with a negligible error when the number of trainable diffractive elements/neurons (*N*) approaches *N*_*i*_*N*_*o*_, where *N*_*i*_ and *N*_*o*_ represent the number of pixels at the input and output FOVs, respectively. In this work, we use polarization multiplexing between the input and output FOVs of a diffractive network to increase the capacity of diffractive computing and all-optically perform a group of arbitrary linear transformations that are complex-valued. These polarization-multiplexed diffractive network designs are *not* based on birefringent, anisotropic or polarization-sensitive materials; instead, our designs utilize standard diffractive surfaces where the phase and amplitude transmission coefficients of each trainable diffractive feature are independent of the polarization state of the input light. Using a network design solely based on standard isotropic diffractive materials makes our designs simpler in terms of material selection, fabrication and scale-up; however, it also makes the diffractive network insensitive to different polarization states, and therefore, polarization-multiplexed all-optical computation of different transformations becomes impossible. To overcome this challenge, we used a non-trainable, pre-determined array of linear polarizers (at 0°, 45°, 90° and 135°) within the diffractive network that acted as polarization seeds for the trainable isotropic diffractive layers to all-optically execute different linear transformations through input/output polarization multiplexing (see Fig. 1a). Stated differently, we used data-driven training and optimization of isotropic diffractive layers to encode different linear transformations into different input/output polarization combinations, and this encoding is made possible by the polarization mode diversity introduced by a non-trainable, pre-determined array of linear polarizers within the diffractive volume.

**Fig. 2:**
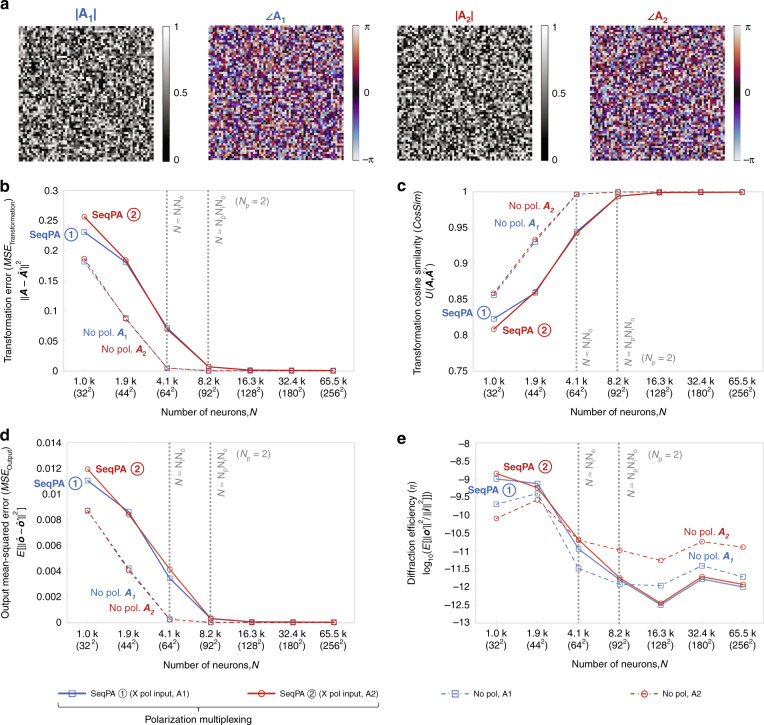
Diffractive all-optical transformation results for 2-channel polarization multiplexing using the sequential polarization access (SeqPA) mode. **a** Amplitude and phase of the arbitrarily generated matrices ***A***_**1**_ and ***A***_**2**_, which serve as the ground truth (target) for the diffractive all-optical transformations. **b** Curves representing the normalized mean-squared error between the ground truth transformation matrices (***A***_**1**_ and ***A***_**2**_) and the all-optical transforms (***A***_1_^′^ and ***A***_2_^′^) resulting from the trained diffractive networks as a function of the number of diffractive neurons *N*. The solid curves are achieved by the polarization-multiplexed diffractive networks trained using the SeqPA mode, which are compared with the dashed curves achieved by the regular diffractive networks trained with the same set of *N* but without any polarization multiplexing. For the polarization-multiplexed models, the results for the two polarization channels ① and ②, corresponding to transforms ***A***′_1_ and ***A***′_2_, are shown in separate curves that are labeled with “SeqPA ①” and “SeqPA ②”, respectively. For the regular diffractive models without polarization multiplexing, the results for all-optical approximation of ***A***_**1**_ and ***A***_**2**_ are shown in separate curves labeled with “No pol. ***A***_**1**_” and “No pol. ***A***_**2**_”, respectively. The space between the simulation data points is linearly interpolated. **c** Same as (**b**) but the cosine similarity between the all-optical transforms and their ground truth shown in (**a**) is reported. **d** Same as (**b**) but the mean-squared error between the diffractive network output fields and their ground truth is reported. **e** Diffraction efficiency of the presented diffractive networks

**Fig. 3:**
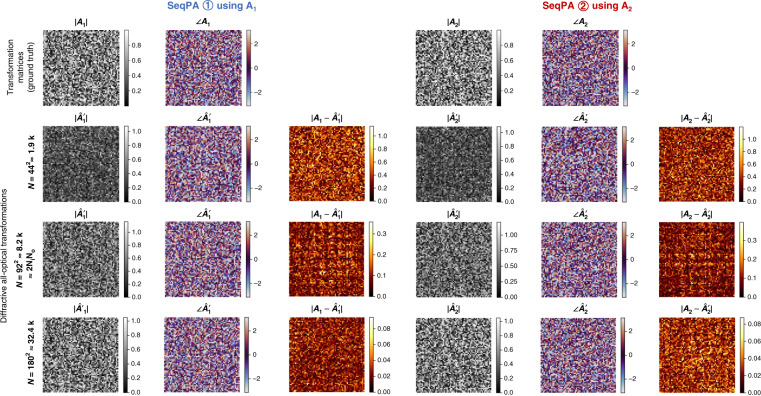
All-optical transformation matrices estimated by the 2-channel polarization multiplexed diffractive designs trained using the SeqPA mode with *N* = 44^2^, 92^2^ and 180^2^. The differences of these matrices from the ground truth matrices are also shown

**Fig. 4:**
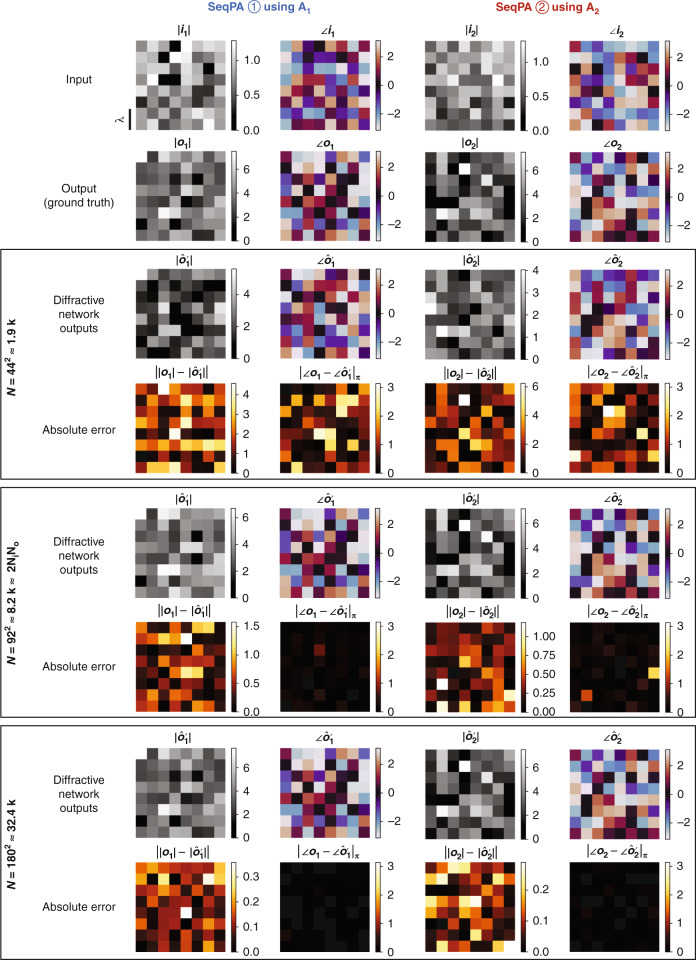
Examples of input/output complex fields for the ground truth transformations presented in Figs. 2 and 3 along with the output fields computed by the 2-channel polarization-multiplexed diffractive designs trained with the SeqPA mode using *N* = 44^2^, 92^2^ and 180^2^. Note that $\left| {\angle {{{\boldsymbol{o}}}} - \angle \widehat {{{{\boldsymbol{o}}}}^\prime }} \right|_\pi$ indicates the wrapped phase difference between the ground truth output field ***o*** and the normalized diffractive network output field $\widehat {{{{\boldsymbol{o}}}}^\prime }$

**Fig. 5:**
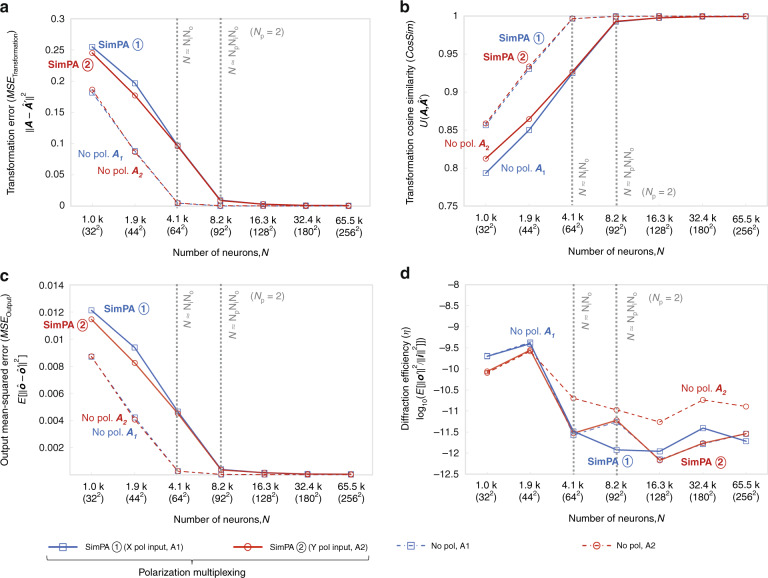
Diffractive all-optical transformation results for 2-channel polarization multiplexing using the simultaneous polarization access (SimPA) mode. **a** Curves representing the normalized mean-squared error between the ground truth transformation matrices (***A***_**1**_ and ***A***_**2**_) and the all-optical transforms (***A***′_1_ and ***A***′_2_) resulting from the trained diffractive networks as a function of the number of diffractive neurons *N*. The solid curves are achieved by the polarization-multiplexed diffractive systems trained using the SimPA mode, which are compared with the dashed curves achieved by the regular diffractive networks trained with the same set of *N* but without any polarization multiplexing. For the polarization-multiplexed models, the results for the two polarization channels ① and ②, corresponding to transforms ***A***′_1_ and ***A***′_2_, are shown in separate curves that are labeled with “SimPA ①” and “SimPA ②”, respectively. For the regular models without polarization multiplexing, the results for all-optical approximation of ***A***_**1**_ and ***A***_**2**_ are shown in separate curves labeled with “No pol. ***A***_**1**_” and “No pol. ***A***_**2**_”, respectively. The space between the simulation data points is linearly interpolated. **b** Same as (**a**) but the cosine similarity between the all-optical transforms and their ground truth is reported. **c** Same as (**a**) but the mean-squared error between the diffractive network output fields and their ground truth is reported. **d** Diffraction efficiency of the presented diffractive networks

In our first implementation, we performed two different, arbitrarily selected linear transformations (i.e., *N*_*p*_ = 2) using a diffractive network composed of four transmissive layers that are jointly optimized using deep learning, where the first target linear transformation was assigned to *x* (0°) linear input and x linear output polarization combination, and the second target linear transformation was assigned to *y* (90°) linear input and y linear output polarization combination. For this case of *N*_*p*_ = 2, there are two different schemes (Fig. 1b) to all-optically access/implement the desired linear transformations: sequential (x and y input polarization states encode the input information sequentially, one after another) or simultaneous (x and y input polarizations encode the input information at the same time within the input FOV). Our numerical results (Figs. 2–5) reveal that one can successfully train a diffractive network under each one of these operation modes (sequential vs. simultaneous) to approximate the two target, arbitrary-selected linear transformations with a negligible error when the number of trainable diffractive neurons *N* approaches $N_pN_iN_o = 2N_iN_o$.

**Fig. 6:**
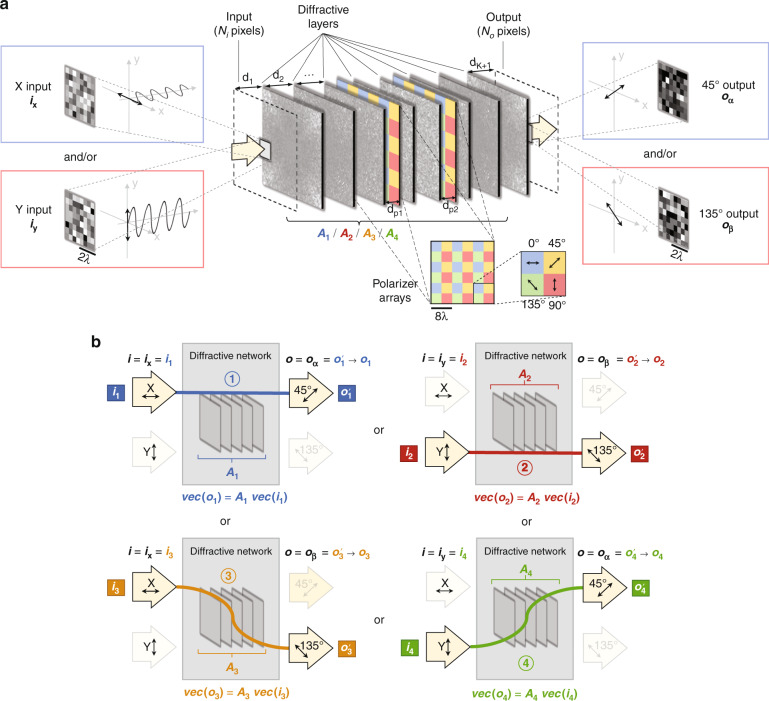
Schematic of 4-channel polarization-multiplexed all-optical diffractive computing framework for performing four unique linear transformations through a single diffractive network. **a** Optical layout of the polarization-encoded diffractive network, where eight trained diffractive layers and two arrays of linear polarizers are jointly used to perform four distinct, complex-valued linear transformations between the input field ***i*** and the output field ***o*** by using polarization encoding/decoding at the input/output FOVs. **b** Schematic for the operation of the 4-channel polarization-multiplexed all-optical computing framework, where the four polarization channels, ①, ②, ③ and ④, are formed by sequentially connecting one of the two input polarization states with one of the two output polarization states

In our second implementation (Fig. 6), we performed four different, arbitrary linear transformations (i.e., *N*_*p*_ = 4) using a diffractive network composed of eight transmissive layers that are jointly optimized using deep learning and examples of input/output fields corresponding to the selected complex-valued linear transformations (ground truth). In this case, the first target transformation was assigned to x linear input and 45° linear output polarization combination, the second target transformation was assigned to y linear input and 135° linear output polarization combination, the third target transformation was assigned to x linear input and 135° linear output polarization combination and finally the fourth target transformation was assigned to y linear input and 45° linear output polarization combination. Our analyses of this 4-channel polarization-multiplexed diffractive system show that when $N \ge N_pN_iN_o = 4N_iN_o$, all the target linear transformations can be successfully approximated, following a similar conclusion as in the first implementation case (*N*_*p*_ = 2).

Without the use of a non-trainable, pre-determined array of linear polarizers acting as polarization seeds within the network, none of these multiplexing results could be achieved using isotropic diffractive materials, no matter how they are trained or optimized, since they would normally perform the same transformation under different input polarization states.

Our results should *not* be confused with polarization-multiplexed (or wavelength/illumination multiplexed) projection of a set of desired complex fields at the output of a metamaterial design; such multiplexed metamaterial systems do not implement an arbitrary matrix multiplication operation. Each input-output polarization combination in our diffractive design represents an all-optical implementation of a unique linear transformation between the input and output FOVs. Therefore, for each input-output polarization combination, infinitely many different target complex fields can be all-optically synthesized by the trained diffractive network in response to different input field distributions; and this capability accurately defines the corresponding complex-valued linear transformation at the output FOV for all the possible and infinitely many combinations of phase and amplitude distributions at the input FOV.

A polarization-multiplexed diffractive network can perform an arbitrary set of target linear transformations using the same diffractive layers that all-optically implement a distinct complex-valued linear transformation at a selected input/output polarization combination. We believe that this unique framework will be valuable in developing high-throughput optical processors and polarization-based machine vision systems operating at different parts of the electromagnetic spectrum. Moreover, the presented diffractive computing platform and the underlying concepts can be used to develop polarization-aware optical information processing systems for e.g., detection, localization, and statistical inference of objects with unique polarization properties.

## Results

Throughout this section, the terms “diffractive optical network,” “diffractive network,” and “diffractive processor” are interchangeably used. The schematic of our framework for 2-channel polarization-multiplexed all-optical computing (*N*_*p*_ = 2) is shown in Fig. 1a. A polarization-encoded diffractive neural network, composed of 4 trainable diffractive layers, is trained to all-optically perform 2 distinct, complex-valued linear transformations between the input and output FOVs through 2 orthogonal polarization channels. The pre-determined polarizer array (which is treated as non-trainable) consists of multiple linear polarizer units with four different polarization directions: 0°, 45°, 90° and 135°. This non-trainable polarizer array is positioned close to the center of the diffractive volume (i.e., between the 2^nd^ and 3^rd^ trainable diffractive layers) so that the resulting polarization modulation does not directly dominate the output field; the former and latter diffractive layers are jointly optimized to effectively communicate with the polarizer array and all-optically implement the desired group of linear transformations. More details about the architecture, optical forward model and training details of the polarization diffractive network can be found in the Methods section.

We use ***i*** and ***o*** to denote the complex-valued, vectorized versions of the 2D input and output complex fields located at the input and output FOVs of the diffractive network, respectively, as presented in Fig. 1a. Based on the scalar diffraction theory, here ***i***_**x**_ and ***o***_**x**_ represent the column vectors of the complex fields generated by sampling the x-polarized optical fields within the input and output FOVs, respectively, and vectorizing the resulting 2D matrices in a column-major order. Similar to ***i***_**x**_ and ***o***_**x**_, ***i***_**y**_ and ***o***_**y**_ are their counterparts generated by sampling the y-polarized optical fields within the input and output FOVs, respectively. Based on this notation, (***i***_**x**_, ***i***_**y**_) and (***o***_**x**_, ***o***_**y**_) can be considered to represent the input and output channels of our polarization-multiplexed diffractive network, respectively. In our analyses, the number of pixels in the input and output FOVs are both taken as *N*_*i*_ = *N*_*o*_ = 8^2^ = 64, such that each target linear transformation matrix has 64^2^ complex-valued entries.

In this first implementation with *N*_*p*_ = 2, we randomly generated two complex-valued matrices ***A***_**1**_ and ***A***_**2**_, each with a size of *N*_*i*_ × *N*_*o*_ = 64^2^, to serve as two unique arbitrary linear transformations that we would like to all-optically implement using a single polarization diffractive network. Visualized in Fig. 2a with their amplitude and phase components, these two matrices are independently generated using different random seeds, and the difference between the two matrices can be found in Fig. S1. We also randomly generated two training sets of complex-valued vectors {***i***_**1**_} and {***i***_**2**_} with *N*_*i*_ = 64 as input fields, and constructed the corresponding sets of output field vectors {***o***_**1**_} and {***o***_**2**_} using ${{{\boldsymbol{o}}}}_1 = {{{\boldsymbol{A}}}}_1{{{\boldsymbol{i}}}}_1$ and ${{{\boldsymbol{o}}}}_2 = {{{\boldsymbol{A}}}}_2{{{\boldsymbol{i}}}}_2$, respectively. For each one of these training sets, {***i***_**1**_} and {***i***_**2**_}, we used 55,000 randomly generated complex fields in our training process. A further increase in the size of this training dataset (to e.g., >100,000 randomly generated complex fields) could improve the transformation approximation accuracy of the trained diffractive networks, but would not change the general conclusions of this manuscript and therefore is left as future work.

Based on the given inputs of {***i***_**1**_} and {***i***_**2**_}, the ultimate goal of training our polarization-multiplexed diffractive network is to simultaneously compute the all-optical output fields {${{{\boldsymbol{o}}}}_1^\prime$} and {${{{\boldsymbol{o}}}}_2^\prime$} to come close to the output ground truth (target) fields {***o***_**1**_} and {***o***_**2**_}; this way, the all-optical transformations ***A***′_1_ and ***A***′_2_ performed by the trained single diffractive system represent an accurate approximation to their ground truth (target) transformation matrices ***A***_**1**_ and ***A***_**2**_. It should be emphasized that we are not aiming to train the diffractive network to implement the correct linear transformations for only a few input-output field pairs. Instead, despite the limited number of input/output field patterns used during the training process, our goal is to generalize to *any* pairs of (***i***_**1**_, ***o***_**1**_) and (***i***_**2**_, ***o***_**2**_) that satisfy ${{{\boldsymbol{o}}}}_1 = {{{\boldsymbol{A}}}}_1{{{\boldsymbol{i}}}}_1$ and ${{{\boldsymbol{o}}}}_2 = {{{\boldsymbol{A}}}}_2{{{\boldsymbol{i}}}}_2$. More details about the training data generation can be found in “Methods”.

To form two unique diffractive information processing pipelines in the same diffractive network for performing the linear transformations given by ***A***_**1**_ and ***A***_**2**_, as shown in Fig. 1a we matched the input fields and the diffractive output pairs, {(***i***_**1**_, ${{{\boldsymbol{o}}}}_1^\prime$)} and {(***i***_**2**_, ${{{\boldsymbol{o}}}}_2^\prime$)}, with the input and output polarization channels of our diffractive system, i.e., ${{{\boldsymbol{i}}}}_{{{\mathbf{x}}}} = {{{\boldsymbol{i}}}}_1$, ${{{\boldsymbol{i}}}}_{{{\mathbf{y}}}} = {{{\boldsymbol{i}}}}_2$, ${{{\boldsymbol{o}}}}_{{{\mathbf{x}}}} = {{{\boldsymbol{o}}}}_1^\prime$ and ${{{\boldsymbol{o}}}}_{{{\mathbf{y}}}} = {{{\boldsymbol{o}}}}_2^\prime$. That is to say, the ***A***′_1_ transformation is performed by encoding the corresponding input field data ***i***_**1**_ into the x-polarized optical field within the input FOV, using e.g., an x-aligned linear polarizer, and decoding (sampling) the x-polarized component of the field within the output FOV as the computed output field ${{{\boldsymbol{o}}}}_1^\prime$ using e.g., an x-polarized analyzer. We denote this diffractive information processing channel as the channel ① in Fig. 1b. It is also a similar case for the ***A***′_2_ transformation, except this time the y polarization is employed at the input and output FOVs, and this diffractive information processing channel is denoted as the channel ②. With this polarization encoding scheme, there are potentially two modes to perform the data inference through the same diffractive network: (1) in two sequential, successive accesses to the diffractive system, each time feeding the input data using its assigned polarization channel, and obtaining the corresponding output (see Fig. 1b, left); and (2) in single access to the diffractive system, by feeding the input data of both of the two polarization channels in parallel, and obtaining the two corresponding outputs simultaneously (see Fig. 1b, right). We term the former and latter approaches as the “sequential polarization access” (SeqPA) mode and the “simultaneous polarization access” (SimPA) mode, respectively. We should emphasize that the fundamental difference between these two modes of operation lies in the input information: the SimPA mode can simultaneously accept both of the input polarization states (e.g., *x* and *y* polarization) for encoding two different channels of input information, while the SeqPA mode can accept a single polarization state as its input so that only one channel of input information is encoded at a given time. Therefore, if the input FOV simultaneously encodes the data to be processed in two different polarization states, or if the time lag caused by switching between different input polarization states is unacceptable (such as e.g., an input FOV that includes a rapidly changing dynamic scene with specific polarization information), then only the SimPA mode would be suitable to process the input encoding. Conversely, if the system is only required to compute a single linear transformation at a given time, or if the time lag caused by switching back and forth between two different input polarization states is acceptable, then SeqPA mode can be used. Detailed analyses of these two modes of operation are presented in the following subsections.

### 2-channel polarization-multiplexed all-optical diffractive computing using the sequential polarization access (SeqPA) mode

As shown in Fig. 1b, left, with the input data ***i***_**1**_ and ***i***_**2**_ being separately and sequentially fed into the polarization channels ① and ②, respectively, the all-optical computed outputs ${{{\boldsymbol{o}}}}_1^\prime$ and ${{{\boldsymbol{o}}}}_2^\prime$ are also collected successively using the same diffractive network hardware. By employing this SeqPA strategy, we trained polarization-multiplexed diffractive networks with different numbers of trainable diffractive neurons, i.e., *N =* {32^2^, 44^2^, 64^2^, 92^2^, 128^2^, 180^2^, 256^2^}, all using the same training datasets {(***i***_**1**_, ***o***_**1**_)} and {(***i***_**2**_, ***o***_**2**_)} and the same number of epochs. To benchmark the performances of these multiplexed diffractive networks, for each transformation dataset and *N*, we also trained regular diffractive networks without the polarizer array or any polarization encoding/decoding at the input/output FOVs, which constitute our baseline. These regular diffractive networks, denoted as “*No pol*.” in our analyses, are trained to approximate *only one* linear transformation (i.e., either ***A***_**1**_ or ***A***_**2**_), and therefore they are referred to as *N*_*p*_ = 1 (no polarization multiplexing).

Figure 2b–e present the quantitative comparison of the all-optical transformation results obtained using the trained diffractive networks described above. Three different metrics were used to quantify the transformation accuracy and generalization performance of these diffractive networks: (1) the normalized transformation mean-squared error (${\rm{MSE}}_{{{{\mathrm{Transformation}}}}}$), (2) the cosine similarity (*CosSim*) between the all-optical transforms and the target transforms, and (3) the mean-squared error between the diffractive network output fields and their ground truth (${\rm{MSE}}_{{{{\mathrm{Output}}}}}$). These performance metrics are reported in Fig. 2b-d, as a function of the number of diffractive neurons (*N*) used in each design. Note that the transformation error of the polarization-multiplexed diffractive systems is calculated per polarization channel. More details about the formulations of these performance metrics can be found in Methods. In Fig. 2b, it can be seen that the transformation errors of all the trained diffractive models monotonically decrease as *N* increases, which is expected due to the increased degrees of freedom in the diffractive processor. In the standard diffractive networks without polarization multiplexing (dash-dotted curves labeled with “No pol. ***A***_**1**_” or “No pol. ***A***_**2**_”), the transformation errors for implementing ***A***_**1**_ or ***A***_**2**_ are almost the same (which indicates that these randomly selected matrices, ***A***_**1**_ and ***A***_**2**_, represent similar computational complexity; also see Fig. S1). The approximation errors of these standard diffractive networks, No pol. ***A***_**1**_ and No pol. ***A***_**2**_, both approach to 0 as *N* approaches $N_iN_o = 64^2 \approx 4.1\,k$. In the polarization-multiplexed diffractive models (solid curves labeled with “SeqPA ①” or “SeqPA ②”), the transformation errors ${\rm{MSE}}_{{{{\mathrm{Transformation}}}}}$ for the two distinct transforms computed through the two polarization channels are also very close to each other for all values of *N*, demonstrating no bias toward any specific polarization channel or transform. The approximation errors of these polarization-multiplexed models approach to 0 as *N* approaches $N_pN_iN_o = 2N_iN_o =$ 92^2^ ≈ 8.2 *k*. This finding indicates that compared with the baseline diffractive models that can only perform a *single* transform, performing two unique transforms using polarization multiplexing through the same diffractive model requires the number of trainable neurons *N* to double. This conclusion is further supported by the results of the other two performance metrics, *CosSim* (Fig. 2c) and ${\rm{MSE}}_{{{{\mathrm{Output}}}}}$ (Fig. 2d) that both show the same trends as in Fig. 2b: for the baseline diffractive models *CosSim* and ${\rm{MSE}}_{{{{\mathrm{Output}}}}}$ approach 1 and 0 as *N* approaches *N*_*i*_*N*_*o*_, respectively, while for the polarization-multiplexed diffractive models, the two metrics approach 1 and 0 as *N* approaches $N_pN_iN_o = 2N_iN_o$. Apart from the metrics that are used to evaluate the transformation performance, we also report the output diffraction efficiencies (*η*) of these diffractive models in Fig. 2e, which reveal that compared with the baseline diffractive networks (*No pol*.), the diffraction efficiencies of the polarization-multiplexed diffractive models trained using the SeqPA mode reach a similar level.

To further demonstrate the performance of our polarization-multiplexed diffractive networks, in Fig. 3 we show examples of the ground truth transformation matrices (i.e., ***A***_**1**_ and ***A***_**2**_) and their counterparts (i.e., ***A***′_1_ and ***A***′_2_) resulting from the diffractive designs with *N* = {44^2^, 92^2^, 180^2^}, along with the amplitude and phase absolute errors. Exemplary complex-valued input-output fields from the same set of diffractive designs are also presented in Fig. 4. Figures 3 and 4 reveal that for both of the polarization channels, when $N \ge N_pN_iN_o = 2N_iN_o$, the all-optical transformation matrices and the output complex fields very well match their ground truth targets with negligible absolute errors, which are also in line with the observations made in Fig. 2.

### 2-channel polarization-multiplexed all-optical diffractive computing using the simultaneous polarization access (SimPA) mode

As an alternative to the sequential polarization access (SeqPA) used earlier, we also explored the use of the simultaneous polarization access (SimPA) mode in our all-optical computing framework. As shown in Fig. 1b, right, in single access to the diffractive system, the input complex-valued data ***i***_**1**_ and ***i***_**2**_ are fed into the polarization channels ① and ②, respectively, and the all-optical diffractive outputs ${{{\boldsymbol{o}}}}_1^\prime$ and ${{{\boldsymbol{o}}}}_2^\prime$ are collected at the same time through two orthogonal polarization states at the output FOV. Before we trained a new polarization-multiplexed diffractive network from scratch using the SimPA mode, we first took our earlier diffractive designs trained using the SeqPA mode and tested them directly using the SimPA mode by inputting both polarization channels ① and ② *at the same time*, deviating from their training scheme, which only used SeqPA. The results of blindly testing the SeqPA-trained diffractive networks under the SimPA mode are shown in Fig. S2, which reveals inference results with significantly higher values of ${\rm{MSE}}_{{{{\mathrm{Transformation}}}}}$ and ${\rm{MSE}}_{{{{\mathrm{Output}}}}}$ and decreased values of *CosSim*, all of which indicate a performance degradation, when we operate a SeqPA-trained diffractive network using the SimPA mode. As shown in Fig. S3, this performance degradation is due to the “cross-talk” between the two transformation channels when both of the input polarization states are at the same time present, which was not considered during the SeqPA-based training process. These results highlight the necessity of training the diffractive system from scratch under the SimPA mode, so that the impact of this cross-talk can be taken into account and minimized during the iterative design process. A related mathematical analysis that supports the same conclusion is reported in Supplementary Note 1.

After training our diffractive models from scratch using the SimPA mode, we report their blind testing results in Fig. 5 using the solid curves labeled with “SimPA ①” and “SimPA ②”. The results of the new diffractive designs trained using the SimPA mode demonstrate the success of all-optically performing two different linear transformations in parallel using polarization multiplexing. Our analysis (Fig. 5) also reveals the same conclusions discussed earlier for the models trained using the SeqPA mode: the all-optical transformation performance of polarization-multiplexed diffractive networks very well match the ground truth, desired transformations as *N* approaches $N_pN_iN_o = 2N_iN_o$. Furthermore, as shown in Fig. 5d, the diffraction efficiencies achieved by the polarization-multiplexed diffractive networks reach a similar level as their baseline counterparts that use the same number of diffractive layers, but without the linear polarizer array.

We further compared the blind testing results of these two different modes of operation (SeqPA vs. SimPA) and performed a cross-talk field analysis (see Fig. S3). We found out that the amount of transformation cross-talk in the diffractive models trained using the SimPA mode (shown in the right column of Fig. S3c, d), is ~300-fold lower when compared with the amplitude values of the cross-talk observed in the diffractive designs trained using the SeqPA mode (shown in the left column of Fig. S3c, d). During the diffractive model training, these cross-talk fields are gradually eliminated (penalized) using the SimPA mode of operation to better approximate the ground truth fields. However, for the diffractive models trained under the SeqPA mode, such cross-talk fields are ignored (i.e., remain non-penalized during the training phase) since the SeqPA operation assumes successive access to the diffractive network, one input polarization state at a time. Stated differently, SeqPA trained diffractive networks successfully approximate the target transformations only when they are tested under the same SeqPA mode of operation, and fail due to the field cross-talk when tested under the SimPA mode.

### 4-channel polarization-multiplexed all-optical diffractive computing

So far, we have demonstrated to perform all-optical computing with 2-channel polarization multiplexing through a single diffractive network. To further exploit the polarization multiplexing capability of this diffractive computing framework, next, we explored a 4-channel polarization-multiplexed design for performing 4 different arbitrarily-selected linear transformations through a single diffractive network (i.e., *N*_*p*_ = 4). Figure 6 illustrates the schematics of this framework. As depicted in Fig. 6b, by sequentially connecting one of the two input polarization states with one of the two output polarization states, four transformation channels, ①, ②, ③ and ④, can be formed to all-optically perform *N*_*p*_ = 4 distinct complex-valued transforms using the same diffractive processor. This 4-channel polarization-multiplexed design operates in a similar way as the SeqPA mode, where the different input data are separately and sequentially fed into different input polarization channels. Using this SeqPA operation mode, our diffractive system can accurately perform 4 different complex-valued linear transformations using the same passive diffractive layers, in a single optical network. For example, when only one polarization state (e.g., ***i***_**x**_) is utilized to encode the input data (i.e., ***i*** = ***i***_**x**_ = ***i***_**1**_ = ***i***_**3**_), we can measure the output field at two orthogonal polarization states and simultaneously read out two computed outputs (i.e., ***o***_***α***_ = ***o***_**1**_ and ***o***_**β**_ = ***o***_**3**_), each corresponding to the result of a uniquely different linear transformation (i.e. ***A***_**1**_ or ***A***_**3**_) computed based on the same input; this capability enables parallel optical information processing through the same polarization-encoded diffractive network. The overall design of this 4-channel diffractive system can be considered to utilize the remaining degrees of freedom in the cross-talk channels of the 2-channel system. Additional analysis that supports the same conclusions can be found in Supplementary Note 1.

It is also worth noting that, compared to the 2-channel polarization-multiplexed system reported earlier, the polarization states for the output field sampling in this 4-channel system are selected to be 45° and 135° linear polarization. This design choice is made to balance out the diffraction efficiencies of the resulting 4 different linear transformations that are all-optically performed by the diffractive network. Stated differently, this design choice introduces symmetry to all the input/output polarization combinations that are each assigned to a different linear transformation. In Fig. 6a, b, we denote the two output fields corresponding to the linear polarization directions at 45° and 135° as ***o***_***α***_ and ***o***_**β**_, respectively.

In the light of our earlier findings that point to the need for more diffractive neurons in the case of *N*_*p*_ = 2 when compared to *N*_*p*_ = 1, here we employed 8 successive trainable diffractive layers to increase our degrees of freedom for *N*_*p*_ = 4 design (see Fig. 6a). Also, compared to the earlier 2-channel polarization-multiplexed design, we included an additional linear polarizer array with the same configuration as before (with polarization orientations of 0°, 45°, 90° and 135°) to further enhance the spatial diversity of polarization modes within the diffractive processor. These two linear polarizer arrays are positioned after the 3^rd^ and 5^th^ diffractive layers, respectively. Same as the *N*_*p*_ = 2 diffractive designs, these linear polarizer arrays are pre-determined (i.e., non-trainable) and act as “polarization seeds” within the trained diffractive network.

**Fig. 7:**
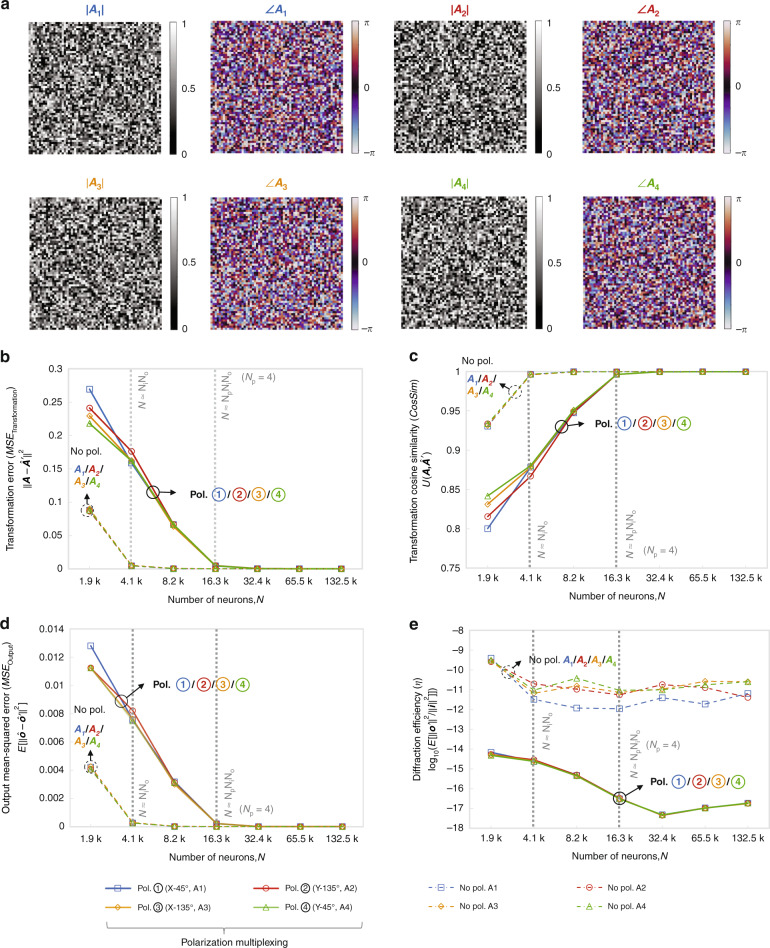
Diffractive all-optical transformation results for 4-channel polarization multiplexing of four distinct arbitrary linear transforms (depicted in Fig. 6). **a** Amplitude and phase of the arbitrarily generated matrices ***A***_**1**_, ***A***_**2**_, ***A***_**3**_ and ***A***_**4**_, which serve as the ground truth (target) for the diffractive all-optical transformations. **b** Curves representing the normalized mean-squared error between the ground truth transformation matrices (***A***_**1**_, ***A***_**2**_, ***A***_**3**_ and ***A***_**4**_) and the all-optical transforms (***A***′_1_, ***A***′_2_, ***A***_3_′and ***A***_4_′, examples shown in Fig. S4) resulting from the trained diffractive networks as a function of the number of diffractive neurons *N*. The solid curves are achieved by the 4-channel polarization-multiplexed diffractive systems, which are compared with the dashed curves achieved by the regular diffractive networks trained with the same set of *N* but without polarization multiplexing. For the polarization-multiplexed models, the results for the four polarization channels ①, ②, ③ and ④ are shown in separate curves but jointly labeled with “Pol. ①/②/③/④” due to the spatial overlap of these curves. For the regular diffractive models without polarization multiplexing, the results for all-optical approximation of ***A***_**1**_, ***A***_**2**_, ***A***_**3**_ and ***A***_**4**_ (individually) are shown in separate curves but jointly labeled with “No pol. ***A***_**1**_/***A***_**2**_/***A***_**3**_/***A***_**4**_” due to the spatial overlap of these curves. The space between the simulation data points is linearly interpolated. **c** Same as (**b**) but cosine similarity between the all-optical transforms and their ground truth is reported. **d** Same as (**b**) but the mean-squared error between the diffractive network output fields and their ground truth is reported. **e** Diffraction efficiency of the presented diffractive networks

Next, we generated random data to train and test our diffractive networks under *N*_*p*_ = 4. In addition to the two randomly generated ground truth transforms ***A***_**1**_ and ***A***_**2**_ that were earlier used for the 2-channel models, we randomly generated two additional complex-valued transforms ***A***_**3**_ and ***A***_**4**_ and accordingly constructed the training and testing dataset consisting of the input and ground truth output fields. These four ground truth (target) transforms are visualized in Fig. 7a, and their differences can be found in Fig. S1. Following the training of the polarization-multiplexed diffractive networks with different *N*, their transformation performance for *N*_*p*_ = 4 is analyzed in Fig. 7b–d based on the same set of performance metrics that were used earlier. These results reveal that, when *N* approaches $N_pN_iN_o = 4N_iN_o = 16.4\,\,k$, the ${\rm{MSE}}_{{{{\mathrm{Transformation}}}}}$ and ${\rm{MSE}}_{{{{\mathrm{Output}}}}}$ of all the four diffractive transformations approach 0, while the *CosSim* approaches 1, demonstrating that all the target linear transformations (***A***_**1**_, ***A***_**2**_, ***A***_**3**_ and ***A***_**4**_) can be successfully approximated by a single diffractive processor with a negligible error if $N \ge N_pN_iN_o$. This is the same conclusion that was reached earlier for *N*_*p*_ = 2.

To further demonstrate the success of these 4-channel polarization-multiplexed diffractive systems, in Fig. S4 we present the ground truth transformation matrices (i.e., ***A***_**1**_, ***A***_**2**_, ***A***_**3**_ and ***A***_**4**_) and their diffractive counterparts (i.e., ***A***′_1_, ***A***′_2_, ***A***_3_′ and ***A***_4_′) designed with *N* = {14.3k, 66.5k}, along with the amplitude and phase errors made in each case. Furthermore, exemplary complex-valued output fields achieved by these diffractive systems are also shown in Fig. S5, all of which confirm the success of the presented 4-channel polarization-multiplexed diffractive designs. Finally, we also analyzed the output diffraction efficiencies of these diffractive models, reported in Fig. 7e. The results show that, compared to their counterparts without polarization encoding (*N*_*p*_ = 1), the polarization-multiplexed diffractive models with *N*_*p*_ = 4 turn out to be less power efficient (per transformation), with an efficiency decrease of ~6 dB at the output FOV. This relatively small difference in the output diffraction efficiencies mainly stems from the different number of diffractive layers used in these two systems: the baseline diffractive systems without polarization encoding use 4 diffractive layers, whereas the 4-channel polarization-multiplexed systems are much deeper, utilizing 8 diffractive layers. Considering that the optical field within a deeper system with more diffractive layers propagates and spreads over a longer axial distance, it exhibits a relatively lower diffraction efficiency. Therefore, these results do not contradict our previous conclusion that the diffraction efficiency of the polarization-multiplexed diffractive network is similar to that of the baseline diffractive system when using the same number of diffractive layers.

Our results and analyses presented so far demonstrated that a single polarization-multiplexed diffractive network can all-optically compute four different complex-valued, arbitrarily-selected linear transformations between its input and output FOVs by using orthogonal linear polarization states. In addition to linear polarization, other polarization states can also be used, without loss of generality, to perform the same multiplexed computational tasks. To demonstrate this capability, we used two orthogonal circular polarization states (i.e., left- and right-hand circular polarization) at the input of a polarization-multiplexed diffractive network to encode the input information; the output channels in this case included x and y linear polarization states, i.e., the 4 different, arbitrarily-selected linear transformations were each assigned to one combination of circular-linear polarization. Our results, reported in Fig. S8, revealed that circular input polarization-multiplexed diffractive processors successfully approximated the target, complex-valued linear transformations, when *N* approaches $N_pN_iN_o = 4N_iN_o = 16.4\,\,k$, arriving at the same conclusion that we had for linear input polarization states. In this diffractive design, we used the same linear polarizer array (i.e., the seed) within the diffractive network volume to communicate between the circular polarization states at the input FOV and the linear polarization states at the output FOV, all-optically performing 4 different complex-valued transformations through the same diffractive network. A mathematical analysis of this design and its relationship to earlier diffractive designs with linear input/output polarization states is also provided in Supplementary Note 1. Since any arbitrary polarization state can be expressed through a superposition of orthogonal linear or circular polarization states, the same diffractive design can be extended to different input/output combinations of other polarization states. As detailed in Supplementary Note 1, a polarization-multiplexed diffractive processor with *N*_*p*_ = 4 can be designed by using input-output combinations of 2 orthogonal input polarization states (e.g., linear, circular or elliptical) and 2 orthogonal output polarization states (e.g., linear, circular or elliptical), where each input-output polarization combination all-optically performs one of the target complex-valued transformations (***A***_**1**_, ***A***_**2**_, ***A***_**3**_, ***A***_**4**_). Supplementary Note 1 further proves that any additional transformation matrix ***A***_***a***_ that can be assigned to a new combination of input-output polarization states of the diffractive network can be written as a linear combination of ***A***_**1**_, ***A***_**2**_, ***A***_**3**_ and ***A***_**4**_.

## Discussion

Our results and analysis demonstrated that, using polarization multiplexing in a single diffractive network, one can all-optically perform a group of complex-valued arbitrary linear transformations at the same output FOV of the diffractive network. In practical applications, these different transformations can cover, for example, various machine vision tasks, such as detection, classification, and localization of objects, which can be programmed into different input/output polarization states. These different tasks could potentially be also performed by employing multiple, separately-optimized diffractive networks, each of which is dedicated to performing a single computational task. However, such an approach would require the precise optical projection of an input FOV (while preserving its phase and amplitude distribution and polarization information) onto separately positioned, individual diffractive networks, and would naturally suffer from additional optical losses and aberrations, misalignment issues, a much larger device footprint and higher manufacturing/alignment-related costs. In contrast, integrating multiple tasks to be all-optically performed within the same diffractive network and a common input FOV provides a much simpler and better design, offering unique advantages such as e.g., speed, compactness, resilience to misalignments and aberrations, power efficiency and cost-effectiveness.

Also note that, it is not practical to spatially superimpose multiple diffractive subsystems, each one separately designed for a unique transformation, using e.g., phase-composite metasurfaces or other metamaterials to create a polarization-multiplexed diffractive processor. First, in the design of each diffractive meta-unit, the cross-talk between the meta-atoms for the two orthogonal polarization states cannot be neglected. Therefore, the direct superposition of two or more different metasurface designs separately trained/designed for each one of the complex transformations would not work due to the cross-talk between the polarization channels of different metasurface designs. Stated differently, different metasurface designs, when put together in order to achieve multiplexed linear transformations in the same optical unit, will fail each other’s transformation accuracy. In addition to this, there will be field cross-talk between the adjacent meta-units that are merged together on the same layer due to the in-plane propagating waves. Although increasing the lateral distance between two adjacent meta-units (from different designs, each targeting one transformation) can weaken the impact of this field cross-talk problem, it will then lead to lower diffraction efficiencies at the output and sacrifice the lateral density of the meta-units at each diffractive layer, thus degrading the computational performance and accuracy of the system. Furthermore, the desired phase response of such polarization-encoded meta-units in general covers a small angular range, leading to a low numerical aperture (NA) that fundamentally limits the connectivity between the diffractive layers. In our diffractive solutions, each isotropic feature of our diffractive network communicates with the following diffractive layer(s) with an NA of *n* (*n* = 1 in air). However, metasurface-based designs would fall short to offer such high numerical apertures, because the high spatial frequency components for the orthogonal input polarizations would deviate from the ideal phase response of the meta-unit, introducing errors to the multiplexed linear transformations that are targeted. Due to some of these challenges outlined above, metasurface or metamaterial-based diffractive surfaces have not yet been demonstrated as a solution to universal, all-optical implementation of an arbitrary linear transformation or a group of transformations.

In addition to polarization multiplexing, we should note that other degrees of freedom can be used to implement multiple computational tasks through a single diffractive network. For example, one can divide the input/output FOVs of the diffractive network into multiple regions, where each region is assigned to a unique computing task through spatial-division multiplexing. It is also possible to achieve wavelength-division multiplexing by assigning different wavelengths or spectral bands to independent computing tasks and employing dispersive elements in the diffractive computing system. In contrast to these other possible methods of information multiplexing, the polarization-based multiplexing that we reported here requires solely the addition of linear polarizers to a diffractive network without changing its architecture. Such polarizers are readily available (e.g., polarizing films), even integrated with the individual pixels of polarization-based imaging systems^60^, and can be adapted to a wide range of wavelengths. Furthermore, polarization multiplexing can be flexibly coupled with other multiplexing methods (such as spectral and/or spatial multiplexing) to further increase the computing capacity of the diffractive network.

Unlike the diffractive layers, where the transmission coefficients are trained and optimized to all-optically perform the target transformations, the design and arrangement of the seed polarizer arrays between the diffractive layers are treated as hyperparameters that are pre-determined and non-trainable. Therefore, the parameters of the embedded polarizers including their number, size, and orientation are fixed during the training process. The polarization modulation induced by these polarizer arrays remains unchanged and was not used as learnable degrees of freedom for our diffractive computing system to approximate the target transformations. Furthermore, their total number is small, i.e., we only used 6 × 6 = 36 linear polarizers per array, which is negligible when compared to *N*. An increase in the number of linear polarizers per plane would not improve the approximation power of our diffractive network to perform arbitrary linear transformations. However, the topology of such polarizer seeds could potentially impact the performance of our polarization-multiplexed diffractive computing system. To explore this, we adjusted several key parameters of the linear polarizer array used in our diffractive processor designs including e.g., 1) the period of each polarizer unit, 2) the overall size of each polarizer array, and 3) the number and position of the polarizer arrays within the diffractive network. For this comparative analysis we used as our test-bed the 4-channel polarization-multiplexed diffractive system with $N = N_pN_iN_o = 16.3\,\,k$ and the same complex-valued target linear transforms (i.e., ***A***_**1**_, ***A***_**2**_, ***A***_**3**_ and ***A***_**4**_), the results of which are summarized in Supplementary Note 2. Based on these analyses, we observe that: (1) a better approximation accuracy can be achieved when the period of each linear polarization unit on the polarizer array is ≤4*λ*, and a period of ~4*λ* empirically appears as an optimal choice, also providing an improved output diffraction efficiency (see Supplementary Fig. SN2); (2) the linear transformation accuracy and the diffraction efficiency of the system can be optimized by using polarizer arrays with a sufficiently large size, i.e., at least matching the size of the neighboring diffractive layers; (3) using two polarizer arrays and placing them apart with an axial distance of ~8*λ* within the diffractive volume can provide improved results for the all-optical transformation accuracy and diffraction efficiency of *N*_*p*_ = 4 designs; and (4) using too many (e.g., >6) polarizer arrays within a diffractive network can lead to severe degradation in the computational accuracy of the system (unless more diffractive layers are added to the design).

We would like to also emphasize that the reported polarization-multiplexed diffractive networks can be directly applied to 2D arrays of phase and amplitude input data. Compared to other optical computing systems operating based on e.g., integrated photonics, which requires 1D inputs and phase recovery if the information is represented in the phase channel, the capability to directly process and analyze raw 2D complex fields makes our framework highly advantageous for visual computing tasks. On the other hand, unless spatial light modulators (SLMs) are employed as part of the diffractive system (see e.g., the Supplementary Information of ref. ^23^. for a discussion on reconfigurable networks), each physically fabricated diffractive network is fixed and would need to be retrained and fabricated again as the target transformations change, which is a limitation of *passive* diffractive systems.

There are additional limitations of the presented diffractive computing framework. First, polarization-multiplexed diffractive computing systems present lower diffraction efficiencies at their output FOV compared to regular diffractive networks without polarization multiplexing (see Figs. 2e and 5d). Several remedies can be used to improve the output diffraction efficiency such as e.g., adding a diffraction-efficiency-related penalty term to the training loss function, and/or restricting the diffractive layers to perform phase-only modulation. The efficacy of using these approaches in a regular diffractive network design (without polarization multiplexing) to improve the output diffraction efficiency has already been demonstrated in our earlier work^69^. To exemplify the performance of a phase-only diffractive design and how it can be used to improve the output diffraction efficiency, we trained phase-only diffractive networks from scratch for the 4-channel polarization multiplexing case (*N*_*p*_ = 4), the results of which are summarized in Fig. S6. This analysis revealed that phase-only diffractive designs can achieve significantly better output diffraction efficiencies (improved on average by ~12 dB), while still successfully approximating the target linear transformations (***A***_**1**_, ***A***_**2**_, ***A***_**3**_ and ***A***_**4**_). As a trade-off, however, these phase-only diffractive designs also exhibit reduced degrees of freedom compared to their complex-valued counterparts. As a result of this, we observed that all the target linear transformations were successfully approximated by a single phase-only diffractive processor when *N* approached $2N_pN_iN_o = 8N_iN_o$. This 2-fold “threshold increase” in the number of diffractive features (i.e., $2N_pN_iN_o$ vs. $N_pN_iN_o$) is a direct reflection of the reduced number of trainable transmission parameters per diffractive layer due to the phase-only operation, which is a limitation of phase-only diffractive networks, despite their enhanced output diffraction efficiency. To further validate this conclusion, we also selected another set of 4 target linear transformations by changing the matrix elements to be real-valued, and used them as ground truth to train phase-only polarization-multiplexed diffractive networks with *N*_*p*_ = 4. As shown in Fig. S7, our results reveal that these phase-only diffractive networks can successfully approximate the real-valued target linear transforms when $N \ge N_pN_iN_o = 4N_iN_o$, demonstrating a similar approximation performance, with significantly higher output diffraction efficiency compared to their complex-valued diffractive counterparts. These findings emphasize the value of phase-only diffractive network designs as a photon-efficient solution in polarization-multiplexed diffractive computing, also providing an important rationale for planning the diffractive neuron budget (*N*) for a given computational task.

Other practical concerns that need to be discussed include the potential fabrication and alignment errors, surface reflections, material absorption and non-ideal polarization modulation within the diffractive network, which may altogether limit the performance and accuracy of diffractive computing. Some of these errors can be mitigated by selecting appropriate fabrication methods, e.g., high-precision lithography, and using less absorptive materials. Moreover, our previous results^23,38,44,49,50^ showed that some of these uncontrolled physical errors and imperfections did not lead to a significant discrepancy between the experimental and numerical, expected results, indicating the correctness of the assumptions involved in our optical forward model and training procedures. Even if these errors and imperfections become considerable, the performance degradation of a diffractive network caused by some of these experimental factors can be compensated by incorporating them as random variables into the physical forward model of the diffractive network during the training process. One example of this has been demonstrated in previous work^36^ where the destructive impact of the lateral and axial misalignments of diffractive layers was mitigated by randomly misaligning the diffractive network during its training process. Following a similar strategy, the imperfect polarization extinction ratio (PER) of the polarizer arrays/seeds can also be included as part of our physical forward model using a modified form of the Jones matrices for linear polarizers. This modeling of imperfect PER of linear polarizers during the training phase can mitigate a potential performance degradation in the computational power of a polarization-multiplexed diffractive processor. Supporting this conclusion, Supplementary Note 3 and Supplementary Fig. SN3 report our mathematical analysis and simulation results for using imperfect linear polarizer arrays/seeds in our diffractive network designs. In the same Supplementary Note 3, we also quantified the overall PER of SimPA-based polarization-multiplexed diffractive designs, considering each diffractive network as a monolithic polarization optical element. Our analysis reveals that the SimPA-based 2-channel polarization-multiplexed diffractive design exhibits a very high PER of >51,000. In fact, such a high PER is expected since the SimPA mode is designed to *simultaneously* perform two different linear transformations using two orthogonal polarization states, and therefore undesired polarization cross-talk at the output field-of-view was penalized during the training phase, successfully leading to a high PER per diffractive network. For the SeqPA mode of operation, however, PER is not a meaningful figure-of-merit since only one orthogonal polarization state is read/measured at a given time due to the sequential access of each target transformation through the diffractive network; stated differently, the SeqPA mode of operation does not penalize the leakage of power into an orthogonal polarization state at the output as it does not impact at all the accuracy of each all-optical transformation that is sequentially performed.

In addition to performing multiple arbitrarily-selected linear transformations through polarization encoding, the presented framework can also be used for polarization-aware optical imaging and sensing tasks. Polarization-based optical imaging has been used in many biomedical applications, such as performing diagnoses of diseases, including gout^59,60,70^, malaria infection^71^, squamous cell carcinoma^72^, and cerebral amyloid^73^. We believe that the presented polarization-multiplexed diffractive computing framework exhibits translational potential for some of these biomedical applications including e.g., the all-optical detection and classification of birefringent crystals in bodily fluids for diagnosing various forms of crystal arthropathy^74^.

In conclusion, we introduced a diffractive network-based all-optical computing framework that can perform multiple complex-valued, arbitrary linear transformations using polarization multiplexing. This framework is very compact; for instance, the system depicted in Fig. 1 has a total length of only 20λ in depth, where λ is the illumination wavelength. Our results show that when the number of diffraction elements/neurons, *N*, in a given diffractive network design approaches $N_pN_iN_o$, a group of *N*_*p*_ arbitrarily-selected linear transforms can be all-optically computed at the output FOV of the network with negligible error. We believe that this polarization-multiplexed diffractive computing framework can be used to build all-optical, passive processors that can execute multiple inference tasks in parallel. We further envision that artificially engineered materials with polarization manipulation capabilities^75–79^ can also be combined with advanced diffractive surface fabrication techniques (e.g., high-precision 3D additive manufacturing and photolithography) to allow the use of our diffractive computing framework in different parts of the electromagnetic spectrum.

## Materials and methods

### Forward model of the polarization-multiplexed diffractive optical network

Using Jones calculus^80^, the complex-valued, polarization-multiplexed electrical field ***E*** at a spatial location ($x_m,y_m,z_m$) can be represented as:

1 $${{{\boldsymbol{E}}}}\left( {x_m,y_m,z_m} \right) = \left[ {\begin{array}{*{20}{c}} {E_{{{\mathrm{x}}}}\left( {x_m,y_m,z_m} \right)} \\ {E_{{{\mathrm{y}}}}\left( {x_m,y_m,z_m} \right)} \end{array}} \right]$$

In our implementation, *E*_x_ and *E*_y_ are computed in parallel throughout the entire diffractive system. Since the trainable diffractive layers are *not* polarization-sensitive, the complex-valued modulation generated by these thin diffractive layers is the same for the two orthogonal polarization states. The diffractive layers are assumed to be thin optical modulation elements, where the *m*^th^ feature on the *k*^th^ diffractive layer at location ($x_m,y_m,z_m$) represents a complex-valued transmission coefficient, *t*^*k*^, given by:

2 $$\begin{array}{*{20}{c}} {t^k\left( {x_m,y_m,z_m} \right) = a^k\left( {x_m,y_m,z_m} \right)\exp \left( {j\phi ^k\left( {x_m,y_m,z_m} \right)} \right)} \end{array}$$

In Eq. 2, *a* and *ϕ* denote the amplitude and phase coefficients, respectively. The amplitude and phase coefficients of the diffractive neurons, *a*^*k*^ and *ϕ*^*k*^ ($k \in \left\{ {1,2, \cdots ,K} \right\}$), are both trainable, with a permitted range of 0 to 1 and 0 to 2π, respectively. Before the training starts, *a*^*k*^ and *ϕ*^*k*^ are randomly initialized with a uniform (*U*) distribution of $U[0,1]$and $U[0,2{{{\mathrm{\pi }}}})$, respectively. For a phase-only diffractive design $a^k = 1$. The size of each diffractive neuron on the transmissive layers and the width of the pixels of the input/output fields are both chosen as *λ*/2.

The diffractive layers are connected to each other by free-space wave propagation, which is modeled through the Rayleigh-Sommerfeld diffraction equation:^23,32^

3 $$w_m^k\left( {x,y,z} \right) = \frac{{z - z_i}}{{r^2}}\left( {\frac{1}{{2\pi r}} + \frac{1}{{j\lambda }}} \right)\exp \left( {\frac{{j2\pi r}}{\lambda }} \right)$$

where $w_m^k\left( {x,y,z,\lambda } \right)$ is the complex-valued field on the *m*^th^ neuron of the *k*^th^ layer at (*x*, *y*, *z*) with a wavelength of *λ*, which can be viewed as a secondary wave generated from the source at $\left( {x_m,y_m,z_m} \right)$; and $r = \sqrt {(x - x_m)^2 + (y - y_m)^2 + (z - z_m)^2}$ and $j = \sqrt { - 1}$. For the *k*^th^ layer (*k* ≥1, treating the input plane as the 0^th^ layer), the modulated optical field $E_p^k$ at location (*x*_*m*_, *y*_*m*_, *z*_*m*_) with a polarization state of *p* ($p \in \left\{ {{{{\mathrm{x}}}},{{{\mathrm{y}}}}} \right\}$) is given by:

4 $$\begin{array}{*{20}{l}} E_p^k\left( {x_m,y_m,z_m} \right) = t^k\left( {x_m,y_m,z_m} \right) \cdot \mathop {\sum}\limits_{n \in S} {E_p^{k - 1}\left( {x_n,y_n,z_n} \right)}\\ \cdot w_m^{k - 1}\left( {x_m,y_m,z_m} \right) \end{array}$$

where *S* denotes all the pixels on the previous diffractive layer. For all the diffractive networks trained in this paper, the axial distances $d_0,d_1,...,d_K$ are all chosen as 4*λ*.

When modeling the polarizer elements in our diffractive system, we used Jones matrices to represent the modulation of the complex field brought by the input polarizer, output analyzer, or the polarizer array at location (*x*, *y*, *z*), the process of which can be written as:

5 $$\begin{array}{*{20}{c}} {{{{\boldsymbol{E}}}}_{{{{\mathrm{out}}}}}(x,y,z) = {{{\boldsymbol{J}}}}_{{{{\mathrm{linear}}}}}(x,y,z){{{\boldsymbol{E}}}}_{{{{\mathrm{in}}}}}(x,y,z)} \end{array}$$

where *E*_in_ and *E*_out_ are the vectors denoting the input and output complex field before and after the polarization modulation, each containing two orthogonal components along the x and y directions, i.e., $${{{\boldsymbol{E}}}}_{{{{\mathrm{out}}}}}(x,y,z) = \left[ {\begin{array}{*{20}{c}} {E_{{{{\mathrm{out}}}},{{{\mathrm{x}}}}}(x,y,z)} \\ {E_{{{{\mathrm{out}}}},{{{\mathrm{y}}}}}(x,y,z)} \end{array}} \right]$$ and $${{{\boldsymbol{E}}}}_{{{{\mathrm{in}}}}}(x,y,z) = \left[ {\begin{array}{*{20}{c}} {E_{{{{\mathrm{in}}}},{{{\mathrm{x}}}}}(x,y,z)} \\ {E_{{{{\mathrm{in}}}},{{{\mathrm{y}}}}}(x,y,z)} \end{array}} \right]$$.${{{\boldsymbol{J}}}}_{{{{\mathrm{linear}}}}}(x,y,z)$ represents the Jones matrix of a linear polarizer element, which is given by:

6 $$\begin{array}{*{20}{l}} {{{\boldsymbol{J}}}}_{{{{\mathrm{linear}}}}}(x,y,z)\\ = \left[ {\begin{array}{*{20}{c}} {\cos ^2\theta (x,y,z)} & {\cos \theta (x,y,z) \sin \theta (x,y,z)} \\ {\sin \theta (x,y,z)\cos \theta (x,y,z)} & {\sin ^2\theta (x,y,z)} \end{array}} \right] \end{array}$$

where $\theta (x,y,z)$ is the angle between the x-axis and the polarizing axis of the linear polarizer located at (*x*, *y*, *z*). For the non-trainable, pre-determined polarizer array that is composed of multiple square-shaped linear polarizers, we used in total 4 types of linear polarizer units with 4 different polarizing axis directions, *θ* ={0, 0.25π, 0.5π, and 0.75π}. As illustrated in Fig. 1a, these 4 different types of linear polarizers are spatially binned to have a 2 × 2 period and repeated with 3 periods in each direction, extending into a square region. The side length of each linear polarizer array is 24*λ*. The residual space surrounding the polarizer array is filled with air, without any polarization modulation. For all the diffractive network designs presented in this paper, the axial distances (i.e., *d*_*p*_, *d*_*p*1_ and *d*_*p*2_) between the pre-determined polarizer arrays and the adjacent diffractive layers in front of them are all empirically chosen as 0; stated differently, each linear polarizer array is attached to the isotropic diffractive layer in front of it.

### Preparation of the linear transformation datasets

In our diffractive network designs, the input and output FOVs have the same size of 8 × 8 pixels, i.e., ${{{\boldsymbol{i}}}}_c,{{{\boldsymbol{o}}}}_c \in {\mathbb C}^{8 \times 8}$ ($c \in \left\{ {1,2,3,4} \right\}$). The size of the transformation matrices is equal to 64 × 64, i.e., ${{{\boldsymbol{A}}}}_c \in {\mathbb C}^{64 \times 64}$ ($c \in \left\{ {1,2,3,4} \right\}$). The amplitude and phase components of the complex-valued transformation matrices ***A***_*c*_ used in this paper were generated with a uniform (*U*) distribution of $U[0,1]$and $U[0,2{{{\mathrm{\pi }}}})$, respectively, using the pseudo-random number generation function *random.uniform()* built-in NumPy. Different random seeds were used to generate these transformation matrices to ensure they were uniquely different (see Fig. S1). Next, the amplitude and phase components of the input fields ***i***_*c*_ ($c \in \left\{ {1,2,3,4} \right\}$) were also randomly generated with a uniform (*U*) distribution of $U[0,1]$ and $U[0,2{{{\mathrm{\pi }}}})$, respectively. The ground truth (target) fields ***o***_*c*_ ($c \in \left\{ {1,2,3,4} \right\}$) were generated by calculating ${{{\boldsymbol{o}}}}_c = {{{\boldsymbol{A}}}}_c{{{\boldsymbol{i}}}}_c$. For each ***A***_*c*_ ($c \in \left\{ {1,2,3,4} \right\}$) we generated a total of 70,000 input/output complex fields to form a dataset, divided into three parts: training, validation, and testing, each containing 55,000, 5,000, and 10,000 complex-valued field pairs, respectively.

### Training loss function

For training of our diffractive networks, we used the mean-squared-error (MSE) loss function, which is defined as:

7 $$\begin{array}{*{20}{c}} {{{{\mathcal{L}}}}_{{{{\mathrm{MSE}}}},c}} & = & {E\left[ {\frac{1}{{N_o}}\mathop {\sum}\limits_{n = 1}^{N_o} {\left| {\widehat {{{{\boldsymbol{o}}}}_1}\left[ n \right] - \widehat {{{{\boldsymbol{o}}}}_1^\prime }\left[ n \right]} \right|^2} } \right]} \\ {} & = & {E\left[ {\frac{1}{{N_o}}\mathop {\sum}\limits_{n = 1}^{N_o} {\left| {\sigma _c{{{\boldsymbol{o}}}}_c\left[ n \right] - \sigma _c^\prime {{{\boldsymbol{o}}}}_c^\prime \left[ n \right]} \right|^2} } \right]} \end{array}$$

where *E*[·] denotes the average across the current batch, *c* stands for the c^th^ polarization channel that is being accessed, and [*n*] indexes the n^th^ element of the vector. *σ*_*c*_ and $\sigma _c^\prime$ are the coefficients used to normalize the energy of the ground truth (target) field ***o***_*c*_ and the diffractive network output field ${{{\boldsymbol{o}}}}_c^\prime$, respectively, which are given by:

8 $$\begin{array}{*{20}{c}} {\sigma _c = \frac{1}{{\sqrt {\mathop {\sum}\nolimits_{n = 1}^{N_o} {\left| {{{{\boldsymbol{o}}}}_c\left[ n \right]} \right|^2} } }}} \end{array}$$

9 $$\begin{array}{*{20}{c}} {\sigma _c^\prime = \frac{{\mathop {\sum }\nolimits_{n = 1}^{N_o} \sigma _c{{{\boldsymbol{o}}}}_c\left[ n \right]{{{\boldsymbol{o}}}}_c^{\prime ^\ast }\left[ n \right]}}{{\mathop {\sum }\nolimits_{n = 1}^{N_o} \left| {{{{\boldsymbol{o}}}}_c^\prime \left[ n \right]} \right|^2}}} \end{array}$$

During the training of the diffractive networks using the SeqPA mode, each polarization channel of the diffractive network is accessed and evaluated cyclically based on the order of the channel number. For instance, for the 2-channel polarization-multiplexed design illustrated in Fig. 1b, left, the access sequence during the training is set to be {①, ②, ①, ②, …}; for the 4-channel polarization-multiplexed design illustrated in Fig. 6, the access sequence is {①, ②, ③, ④, ①, ②, ③, ④, …}. During the access of a certain polarization channel, the diffractive network is fed with one batch of the training input/output complex fields corresponding to the transformation matrix assigned to this channel, and then trained based on the average loss across this batch. Thus, the loss function for training the diffractive designs through the c^th^ polarization channel using the SeqPA mode, ${{{\mathcal{L}}}}_{{{{\mathrm{Seq}}}},c}$, can be simply written as:

10 $$\begin{array}{*{20}{c}} {{{{\mathcal{L}}}}_{{{{\mathrm{Seq}}}},c} = {{{\mathcal{L}}}}_{{{{\mathrm{MSE}}}},c}} \end{array}$$

During the training of the diffractive networks using the SimPA mode, as illustrated in Fig. 1b, right, all the polarization channels of the diffractive network are accessed simultaneously, and the training data are fed into the channels at the same time. For this SimPA mode, the diffractive network is trained based on the loss averaged across the different polarization channels and complex-valued fields in the current batch, where the loss function ${{{\mathcal{L}}}}_{{{{\mathrm{Sim}}}}}$ can be written as:

11 $$\begin{array}{*{20}{c}} {{{{\mathcal{L}}}}_{{{{\mathrm{Sim}}}}} = \frac{1}{{N_p}}\mathop {\sum}\limits_{c = 1}^{N_p} {{{{\mathcal{L}}}}_{{{{\mathrm{MSE}}}},c}} } \end{array}$$

### Performance metrics used for the quantification of all-optical transformation errors

To quantitatively evaluate the transformation results of the polarization-multiplexed diffractive networks, four performance metrics were calculated per polarization channel of the diffractive designs using the testing dataset: (1) the normalized transformation mean-squared error (${\rm{MSE}}_{{{{\mathrm{Transformation}}}}}$), (2) the cosine similarity (*CosSim*) between the all-optical transforms and the target transforms, (3) the normalized mean-squared error between the diffractive network output fields and their ground truth (${\rm{MSE}}_{{{{\mathrm{Output}}}}}$), and (4) the output diffraction efficiency (*η*). The transformation error for the c^th^ polarization channel of the diffractive network, ${\rm{MSE}}_{{{{\mathrm{Transformation}}}},c}$, is defined as:

12 $$\begin{array}{*{20}{c}} {{\rm{MSE}}_{{{{\mathrm{Transformation}}}},c}} & = & {\frac{1}{{N_iN_o}}\mathop {\sum}\limits_{n = 1}^{N_iN_o} {\left| {{{{\boldsymbol{a}}}}_c\left[ n \right] - m_c{{{\boldsymbol{a}}}}_c^\prime \left[ n \right]} \right|^2} } \\ {} & = & {\frac{1}{{N_iN_o}}\mathop {\sum }\limits_{n = 1}^{N_iN_o} \left| {{{{\boldsymbol{a}}}}_c\left[ n \right] - \widehat {{{{\boldsymbol{a}}}}_c^\prime }\left[ n \right]} \right|^2} \end{array}$$

where ***a***_*c*_ is the vectorized version of the ground truth transformation matrix assigned to the c^th^ polarization channel ***A***_*c*_, i.e., ${{{\boldsymbol{a}}}}_c = {{{\mathrm{vec}}}}({{{\boldsymbol{A}}}}_c)$. ${{{\boldsymbol{a}}}}_c^\prime$ are the vectorized version of ${{{\boldsymbol{A}}}}_c^\prime$, which is the all-optical transformation matrix computed using the optimized diffractive transmission coefficients. *m*_*c*_ is a scalar normalization coefficient used to eliminate the effect of diffraction-efficiency related scaling mismatch between ***A***_*c*_ and ${{{\boldsymbol{A}}}}_c^\prime$, i.e.,

13 $$\begin{array}{*{20}{c}} {m_c = \frac{{\mathop {\sum}\nolimits_{n = 1}^{N_iN_o} {{{{\boldsymbol{a}}}}_c\left[ n \right]{{{\boldsymbol{a}}}}_c^{\prime ^\ast }\left[ n \right]} }}{{\mathop {\sum}\nolimits_{n = 1}^{N_iN_o} {\left| {{{{\boldsymbol{a}}}}_c^\prime \left[ n \right]} \right|^2} }}} \end{array}$$

The cosine similarity between the all-optical transform and their target transform for the c^th^ polarization channel, $CosSim_c$, is defined as:

14 $$\begin{array}{*{20}{c}} {CosSim_c = \frac{{\left| {{{{\boldsymbol{a}}}}_c^H{{{\hat{\boldsymbol a}}}}_c^\prime } \right|}}{{\sqrt {\mathop {\sum}\nolimits_{n = 1}^{N_iN_o} {\left| {{{{\boldsymbol{a}}}}_c\left[ n \right]} \right|^2} } \sqrt {\mathop {\sum}\nolimits_{n = 1}^{N_iN_o} {\left| {\widehat {{{{\boldsymbol{a}}}}_c^\prime }\left[ n \right]} \right|^2} } }}} \end{array}$$

The normalized mean-squared error between the diffractive network outputs and their ground truth for the c^th^ polarization channel, ${\rm{MSE}}_{{{{\mathrm{O}}}}utput,c}$, is defined using the same formula as in Eq. 7 (the loss function used during the training process), except for that *E*[·] is calculated across the entire testing set.

The mean diffraction efficiency *η*_*c*_ for the c^th^ polarization channel of the diffractive system is defined as:

15 $$\begin{array}{*{20}{c}} {\eta _c = E\left[ {\frac{{\mathop {\sum}\nolimits_{n = 1}^{N_o} {\left| {{{{\boldsymbol{o}}}}_c^\prime \left[ n \right]} \right|^2} }}{{\mathop {\sum}\nolimits_{n = 1}^{N_i} {\left| {{{{\boldsymbol{i}}}}_c\left[ n \right]} \right|^2} }}} \right]} \end{array}$$

### Training-related details

All the diffractive optical networks used in this work were simulated and trained using Python (v3.8.11) and TensorFlow (v2.6.0, Google Inc.). We selected Adam optimizer^81^ for training all the models, and its parameters were taken as the default values in TensorFlow and kept identical in each model. The batch size and learning rate were set as 8 and 0.001, respectively. The training of the diffractive network models using the SimPA mode was performed with 50 epochs. For training the diffractive models using the SeqPA mode, the 2-channel and 4-channel polarization-multiplexed designs were trained for 100 and 200 epochs, respectively, so that equivalently 50 epochs are dedicated for training each polarization channel of these designs. The best models were selected based on the MSE loss calculated on the validation dataset. For the training of our diffractive models, we used a desktop computer with a GeForce GTX 1080Ti graphical processing unit (GPU, NVidia Inc.) and Intel® Core^TM^ i7-8700 central processing unit (CPU, Intel Inc.) and 64 GB of RAM, running Windows 10 operating system (Microsoft Inc.). The typical time to train a diffractive network model using the SeqPA mode with 2 and 4 polarization channels is ~7 and ~14 h, respectively. The training time for a diffractive model using the SimPA mode with 2 polarization channels is ~4 h.

## Supplementary information

Supplementary Information

## Data Availability

The deep learning models reported in this work used standard libraries and scripts that are publicly available in TensorFlow. All the data and methods needed to evaluate the conclusions of this work are presented in the main text and Supplementary Information. Additional data can be requested from the corresponding author.
