# Supplementary material for: Polarization multiplexed diffractive computing: all-optical implementation of a group of linear transformations through a polarization-encoded diffractive network
Source: Light Sci Appl. 2022 May 26;11:153. doi: 10.1038/s41377-022-00849-x (PMC9133014; doi:10.1038/s41377-022-00849-x)
Supplement: Supplementary file 1 — Supplementary Information [file 41377_2022_849_MOESM1_ESM.pdf]

Polarization Multiplexed Diffractive Computing: All-Optical Implementation of a Group of Linear Transformations Through a Polarization-Encoded Diffractive Network

Supplementary Information

Jingxi Li[1,2,3], Yi-Chun Hung[1], Onur Kulce[1,2,3], Deniz Mengu[1,2,3], and Aydogan Ozcan[1,2,3*]

[1]Electrical and Computer Engineering Department, University of California, Los Angeles, CA, 90095, USA

[2]Bioengineering Department, University of California, Los Angeles, CA, 90095, USA

[3]California NanoSystems Institute (CNSI), University of California, Los Angeles, CA, 90095, USA

[*]Correspondence to: ozcan@ucla.edu

• Supplementary Note 1: Mathematical analysis of different polarization encoding schemes

Here we analyze different polarization encoding schemes that are used in the main text. The optical fields are assumed to be fully polarized.

(1) For the 2-channel polarization multiplexed diffractive system that uses x or y polarization at both its input and output fields-of-view (FOVs), we can describe the transformation relationship between the inputs $\boldsymbol{i}_{\mathrm{x}}$, $\boldsymbol{i}_{\mathrm{y}}$ and the outputs $\boldsymbol{o}_{\mathrm{x}}$, $\boldsymbol{o}_{\mathrm{y}}$ using:

$$\begin{bmatrix}\boldsymbol{o}_{\mathrm{x}}\\ \boldsymbol{o}_{\mathrm{y}}\end{bmatrix} = \begin{bmatrix}\boldsymbol{A}_{x-x} & \boldsymbol{A}_{y-x}\\ \boldsymbol{A}_{x-y} & \boldsymbol{A}_{y-y}\end{bmatrix}\begin{bmatrix}\boldsymbol{i}_{\mathrm{x}}\\ \boldsymbol{i}_{\mathrm{y}}\end{bmatrix} \quad \text{(S1)},$$

where $\boldsymbol{A}_{x-x}$, $\boldsymbol{A}_{y-x}$, $\boldsymbol{A}_{x-y}$ and $\boldsymbol{A}_{y-y}$ are the desired/target transformation matrices represented by our polarization multiplexed diffractive networks.

When a polarization multiplexed diffractive network is operated using the SeqPA mode, we sequentially and separately force $\boldsymbol{i}_{\mathrm{x}} = \boldsymbol{0}$ and $\boldsymbol{i}_{\mathrm{y}} = \boldsymbol{0}$ and read out the corresponding $\boldsymbol{o}_{\mathrm{x}}$ and $\boldsymbol{o}_{\mathrm{y}}$, which can be written as:

$$when\ \ \boldsymbol{i}_{\mathrm{y}} = \boldsymbol{0}, \quad \begin{cases}\boldsymbol{o}_{\mathrm{x}} = \boldsymbol{A}_{x-x}\boldsymbol{i}_{\mathrm{x}}, & (used\ as\ ①)\\ \boldsymbol{o}_{\mathrm{y}} = \boldsymbol{A}_{x-y}\boldsymbol{i}_{\mathrm{x}}, & (not\ used\ in\ SeqPA)\end{cases} \quad \text{(S2)},$$

$$when\ \boldsymbol{i}_{\mathrm{x}} = \boldsymbol{0}, \quad \begin{cases}\boldsymbol{o}_{\mathrm{x}} = \boldsymbol{A}_{y-x}\boldsymbol{i}_{\mathrm{y}}, & (not\ used\ in\ SeqPA)\\ \boldsymbol{o}_{\mathrm{y}} = \boldsymbol{A}_{y-y}\boldsymbol{i}_{\mathrm{y}}, & (used\ as\ ②)\end{cases} \quad \text{(S3)}.$$

So by training the system to approximate $\boldsymbol{o}_{\mathrm{x}} = \boldsymbol{A}_{x-x}\boldsymbol{i}_{\mathrm{x}} \rightarrow \boldsymbol{A}_{\mathbf{1}}\boldsymbol{i}_{\mathrm{x}}$ and $\boldsymbol{o}_{\mathrm{y}} = \boldsymbol{A}_{y-y}\boldsymbol{i}_{\mathrm{y}} \rightarrow \boldsymbol{A}_{\mathbf{2}}\boldsymbol{i}_{\mathrm{y}}$, the diffractive network model converges to satisfy $\boldsymbol{A}'_{\mathbf{1}} = \boldsymbol{A}_{x-x} \rightarrow \boldsymbol{A}_{\mathbf{1}}$ and $\boldsymbol{A}'_{\mathbf{2}} = \boldsymbol{A}_{y-y} \rightarrow \boldsymbol{A}_{\mathbf{2}}$.

When a polarization multiplexed diffractive system is operated using the SimPA mode, where both inputs $\boldsymbol{i}_{\mathrm{x}}$ and $\boldsymbol{i}_{\mathrm{y}}$ are fed into the diffractive networks simultaneously, the system is trained to satisfy

$$\boldsymbol{o}_{\mathrm{x}} = \boldsymbol{A}_{x-x}\boldsymbol{i}_{\mathrm{x}} + \boldsymbol{A}_{y-x}\boldsymbol{i}_{\mathrm{y}} \rightarrow \boldsymbol{A}_{\mathbf{1}}\boldsymbol{i}_{\mathrm{x}} \quad \text{(S4)},$$

$$\boldsymbol{o}_{\mathrm{y}} = \boldsymbol{A}_{x-y}\boldsymbol{i}_{\mathrm{x}} + \boldsymbol{A}_{y-y}\boldsymbol{i}_{\mathrm{y}} \rightarrow \boldsymbol{A}_{\mathbf{2}}\boldsymbol{i}_{\mathrm{y}} \quad \text{(S5)},$$

so that the system converges to approximate $\boldsymbol{A}'_{\mathbf{1}} = \boldsymbol{A}_{x-x} \rightarrow \boldsymbol{A}_{\mathbf{1}}$, $\boldsymbol{A}'_{\mathbf{2}} = \boldsymbol{A}_{y-y} \rightarrow \boldsymbol{A}_{\mathbf{2}}$, and also $\boldsymbol{A}_{y-x}, \boldsymbol{A}_{x-y} \rightarrow \boldsymbol{0}$ (zero cross talk). Therefore, after the training, the transformations performed by the diffractive systems using the SimPA mode can be represented using the following formula:

$$\begin{bmatrix}\boldsymbol{o}_{\mathrm{x}}\\ \boldsymbol{o}_{\mathrm{y}}\end{bmatrix} \approx \begin{bmatrix}\boldsymbol{A}'_{\mathbf{1}} & \boldsymbol{0}\\ \boldsymbol{0} & \boldsymbol{A}'_{\mathbf{2}}\end{bmatrix}\begin{bmatrix}\boldsymbol{i}_{\mathrm{x}}\\ \boldsymbol{i}_{\mathrm{y}}\end{bmatrix} \Leftrightarrow \begin{cases}\boldsymbol{o}_{\mathrm{x}} \approx \boldsymbol{A}'_{\mathbf{1}}\boldsymbol{i}_{\mathrm{x}}, & (used\ as\ ①)\\ \boldsymbol{o}_{\mathrm{y}} \approx \boldsymbol{A}'_{\mathbf{2}}\boldsymbol{i}_{\mathrm{y}}, & (used\ as\ ②)\end{cases} \quad \text{(S6)}.$$

Compared to the SeqPA mode, the SimPA mode presents more constraints to the system (i.e., penalizing $\boldsymbol{A}_{y-x}$ and $\boldsymbol{A}_{x-y}$ to approach $\boldsymbol{0}$). Equation (S6) only holds for the SimPA mode of operation since $\boldsymbol{A}_{y-x}$ and $\boldsymbol{A}_{x-y}$ are not used in sequential polarization access, i.e., are ignored in the SeqPA mode (without any penalties).

(2) For the 4-channel polarization multiplexed diffractive network, the transformation relationship between the inputs $\boldsymbol{i}_{\mathrm{x}}$, $\boldsymbol{i}_{\mathrm{y}}$ and the outputs $\boldsymbol{o}_{\alpha}$, $\boldsymbol{o}_{\beta}$ can be described using the following equation:

$$\begin{bmatrix} \boldsymbol{o}_\alpha \\ \boldsymbol{o}_\beta \end{bmatrix} = \begin{bmatrix} \boldsymbol{A}_{x-\alpha} & \boldsymbol{A}_{y-\alpha} \\ \boldsymbol{A}_{x-\beta} & \boldsymbol{A}_{y-\beta} \end{bmatrix} \begin{bmatrix} \boldsymbol{i}_x \\ \boldsymbol{i}_y \end{bmatrix} \quad \text{(S7)}.$$

We sequentially and separately force $\boldsymbol{i}_\mathrm{x} = \boldsymbol{0}$ or $\boldsymbol{i}_\mathrm{y} = \boldsymbol{0}$ and read out the corresponding $\boldsymbol{o}_\alpha$ and $\boldsymbol{o}_\beta$, which can be written as:

$$\text{when } \boldsymbol{i}_\mathrm{y} = \boldsymbol{0}, \quad \begin{cases} \boldsymbol{o}_\alpha = \boldsymbol{A}_{x-\alpha}\boldsymbol{i}_\mathrm{x}, & (\text{used as } ①) \\ \boldsymbol{o}_\beta = \boldsymbol{A}_{x-\beta}\boldsymbol{i}_\mathrm{x}, & (\text{used as } ③) \end{cases} \quad \text{(S8)},$$

$$\text{when } \boldsymbol{i}_\mathrm{x} = \boldsymbol{0}, \quad \begin{cases} \boldsymbol{o}_\alpha = \boldsymbol{A}_{y-\alpha}\boldsymbol{i}_\mathrm{y}, & (\text{used as } ④) \\ \boldsymbol{o}_\beta = \boldsymbol{A}_{y-\beta}\boldsymbol{i}_\mathrm{y}, & (\text{used as } ②) \end{cases} \quad \text{(S9)}.$$

By training the system to approximate $\boldsymbol{o}_\alpha = \boldsymbol{A}_{x-\alpha}\boldsymbol{i}_\mathrm{x} \rightarrow \boldsymbol{A}_1\boldsymbol{i}_\mathrm{x}$, $\boldsymbol{o}_\beta = \boldsymbol{A}_{y-\beta}\boldsymbol{i}_\mathrm{y} \rightarrow \boldsymbol{A}_2\boldsymbol{i}_\mathrm{y}$, $\boldsymbol{o}_\beta = \boldsymbol{A}_{x-\beta}\boldsymbol{i}_\mathrm{x} \rightarrow \boldsymbol{A}_3\boldsymbol{i}_\mathrm{x}$ and $\boldsymbol{o}_\alpha = \boldsymbol{A}_{y-\alpha}\boldsymbol{i}_\mathrm{y} \rightarrow \boldsymbol{A}_4\boldsymbol{i}_\mathrm{y}$, the diffractive network model converges to satisfy $\boldsymbol{A}'_1 = \boldsymbol{A}_{x-\alpha} \rightarrow \boldsymbol{A}_1$, $\boldsymbol{A}'_2 = \boldsymbol{A}_{y-\beta} \rightarrow \boldsymbol{A}_2$, $\boldsymbol{A}'_3 = \boldsymbol{A}_{x-\beta} \rightarrow \boldsymbol{A}_3$ and $\boldsymbol{A}'_4 = \boldsymbol{A}_{y-\alpha} \rightarrow \boldsymbol{A}_4$. To directly access the four diffractive linear transformation matrices (i.e., $\boldsymbol{A}'_1$, $\boldsymbol{A}'_2$, $\boldsymbol{A}'_3$ and $\boldsymbol{A}'_4$), we set either $\boldsymbol{i}_\mathrm{x}$ or $\boldsymbol{i}_\mathrm{y}$ as 0 and read the output fields of $\boldsymbol{o}_\alpha$ and $\boldsymbol{o}_\beta$ in each case (based on Eqs. (S8-S9)).

(3) The input polarization channels of our diffractive network can also use left and right-hand circular polarization states (LHCP and RHCP):

$$\begin{bmatrix} \boldsymbol{o}_\mathrm{x} \\ \boldsymbol{o}_\mathrm{y} \end{bmatrix} = \begin{bmatrix} \boldsymbol{A}_{LHCP-x} & \boldsymbol{A}_{LHCP-y} \\ \boldsymbol{A}_{RHCP-x} & \boldsymbol{A}_{RHCP-y} \end{bmatrix} \begin{bmatrix} \boldsymbol{i}_{LHCP} \\ \boldsymbol{i}_{RHCP} \end{bmatrix} = \begin{bmatrix} \boldsymbol{A}_{LHCP-x} & \boldsymbol{A}_{LHCP-y} \\ \boldsymbol{A}_{RHCP-x} & \boldsymbol{A}_{RHCP-y} \end{bmatrix} \begin{bmatrix} \boldsymbol{1} & \boldsymbol{e}^{j\pi/2} \\ \boldsymbol{e}^{-j\pi/2} & \boldsymbol{1} \end{bmatrix} \begin{bmatrix} \boldsymbol{i}_x \\ \boldsymbol{i}_y \end{bmatrix}$$

$$= \begin{bmatrix} \boldsymbol{A}_{LHCP-x} - j\boldsymbol{A}_{LHCP-y} & j\boldsymbol{A}_{LHCP-x} + \boldsymbol{A}_{LHCP-y} \\ \boldsymbol{A}_{RHCP-x} - j\boldsymbol{A}_{RHCP-y} & j\boldsymbol{A}_{RHCP-x} + \boldsymbol{A}_{RHCP-y} \end{bmatrix} \begin{bmatrix} \boldsymbol{i}_x \\ \boldsymbol{i}_y \end{bmatrix} \quad \text{(S10)}.$$

Based on Equation (S10), if we use LHCP and RHCP as the input polarization channels of our system, the four linear transformation matrices that are encoded through the same diffractive network can be represented by their counterparts measured using the x and y polarization channels. Also see Supplementary Fig. S8, which reveals that circular input polarization-multiplexed diffractive processors can successfully approximate the target, complex-valued linear transformations, when N approaches $N_pN_iN_o = 4N_iN_o = 16.4k$, arriving at the same conclusion that we had for linear input polarization states. Since this type of Jones unitary transformations can be applied between any combinations of orthogonal polarization states, it can be further inferred that a polarization multiplexed diffractive processor with $N_p = 4$ can be designed by using input-output combinations of 2 orthogonal input polarization states (e.g., linear, circular or elliptical) and 2 orthogonal output polarization states (e.g., linear, circular or elliptical), where each input-output polarization combination all-optically performs one of the target complex-valued linear transformations ($\boldsymbol{A}_1$, $\boldsymbol{A}_2$, $\boldsymbol{A}_3$, $\boldsymbol{A}_4$).

(4) Here, we will explore the case of $N_p > 4$ under the SeqPA mode of operation. Suppose that there exists a new linear transformation $\boldsymbol{A}_{\delta-\gamma}$ independent of the other four target linear transformations ($\boldsymbol{A}_1$, $\boldsymbol{A}_2$, $\boldsymbol{A}_3$, $\boldsymbol{A}_4$). $\boldsymbol{A}_{\delta-\gamma}$ operates between $\boldsymbol{i}_\delta$ and $\boldsymbol{o}_\gamma$ at the input and output FOVs of our polarization multiplexed diffractive system (i.e., $\boldsymbol{i}_\delta$ and $\boldsymbol{o}_\gamma$ are defined by the optical fields linearly polarized at the angles of δ and γ, respectively, from the x-axis):

$$\boldsymbol{o}_\gamma \equiv \boldsymbol{A}_{\delta-\gamma}\boldsymbol{i}_\delta \quad \text{(S11)}.$$

Since $\boldsymbol{i_\delta}$ and $\boldsymbol{o_\gamma}$ share the same input and output FOVs as ($\boldsymbol{i_x}$, $\boldsymbol{i_y}$) and ($\boldsymbol{o_x}$, $\boldsymbol{o_y}$), we can decompose them into x and y polarization states:

$$\boldsymbol{o_\gamma} = cos\gamma\boldsymbol{o_x} + sin\gamma\boldsymbol{o_y} \qquad \text{(S12)},$$

$$\boldsymbol{i_\delta} = cos\delta\boldsymbol{i_x} + sin\delta\boldsymbol{i_y}$$
$$\boldsymbol{i_y} = tan\delta\boldsymbol{i_x} \qquad \text{(S13)}$$

Based on these we can write:

$$\boldsymbol{o_\gamma} = \boldsymbol{A_{\delta-\gamma}}\boldsymbol{i_\delta} = cos\delta\boldsymbol{A_{\delta-\gamma}}\boldsymbol{i_x} + sin\delta\boldsymbol{A_{\delta-\gamma}}\boldsymbol{i_y}$$
$$= \left(cos\delta\boldsymbol{A_{\delta-\gamma}} + tan\delta sin\delta\boldsymbol{A_{\delta-\gamma}}\right)\boldsymbol{i_x} = \boldsymbol{A_{\delta-\gamma}}\boldsymbol{i_x}/cos\delta \qquad \text{(S14)}$$

$$\boldsymbol{o_\gamma} = cos\gamma\left(\boldsymbol{A_{x-x}}\boldsymbol{i_x} + \boldsymbol{A_{y-x}}\boldsymbol{i_y}\right) + sin\gamma\left(\boldsymbol{A_{x-y}}\boldsymbol{i_x} + \boldsymbol{A_{y-y}}\boldsymbol{i_y}\right)$$
$$= \left(cos\gamma\boldsymbol{A_{x-x}} + sin\gamma\boldsymbol{A_{x-y}}\right)\boldsymbol{i_x} + \left(cos\gamma\boldsymbol{A_{y-x}} + sin\gamma\boldsymbol{A_{y-y}}\right)\boldsymbol{i_y}$$
$$= \left(cos\gamma\boldsymbol{A_{x-x}} + sin\gamma\boldsymbol{A_{x-y}} + tan\delta\left(cos\gamma\boldsymbol{A_{y-x}} + sin\gamma\boldsymbol{A_{y-y}}\right)\right)\boldsymbol{i_x} \qquad \text{(S15)}$$

Using Eqs. (S14) and (S15), we can write:

$$\boldsymbol{A_{\delta-\gamma}} = cos\delta\left(cos\gamma\boldsymbol{A_{x-x}} + sin\gamma\boldsymbol{A_{x-y}}\right) + sin\delta\left(cos\gamma\boldsymbol{A_{y-x}} + sin\gamma\boldsymbol{A_{y-y}}\right) \qquad \text{(S16)},$$

Based on Eq. (S16), $\boldsymbol{A_{\delta-\gamma}}$ cannot represent an independent linear transformation, and it linearly depends on $\boldsymbol{A_{x-x}}$, $\boldsymbol{A_{y-x}}$, $\boldsymbol{A_{x-y}}$ and $\boldsymbol{A_{y-y}}$ of the polarization multiplexed diffractive network. Stated differently, an additional transformation matrix $\boldsymbol{A_a} = \boldsymbol{A_{\delta-\gamma}}$ that can be assigned to a new combination of input-output polarization states of the diffractive network can be written as a linear combination of $\boldsymbol{A_1}$, $\boldsymbol{A_2}$, $\boldsymbol{A_3}$ and $\boldsymbol{A_4}$. In this case, depending on the target linear transformation set ($\boldsymbol{A_1}$, $\boldsymbol{A_2}$, $\boldsymbol{A_3}$, $\boldsymbol{A_4}$, $\boldsymbol{A_a}$) and the acceptable error threshold for each transformation, a diffractive design with $N_p > 4$ can be optimized using polarization multiplexing to provide *approximate* solutions to the target linear transformations. However, the approximation error and the computational accuracy for this case of $N_p > 4$ will depend on the Euclidean distances among the target complex-valued transformation matrices, and for certain sets of target linear transformations, the trained diffractive network might fail to achieve an acceptable error threshold in its approximation of $\boldsymbol{A_1}$, $\boldsymbol{A_2}$, $\boldsymbol{A_3}$, $\boldsymbol{A_4}$ and $\boldsymbol{A_a}$.

• Supplementary Note 2: Analysis of the impact of the polarizer array parameters on the computational performance of polarization multiplexed diffractive networks

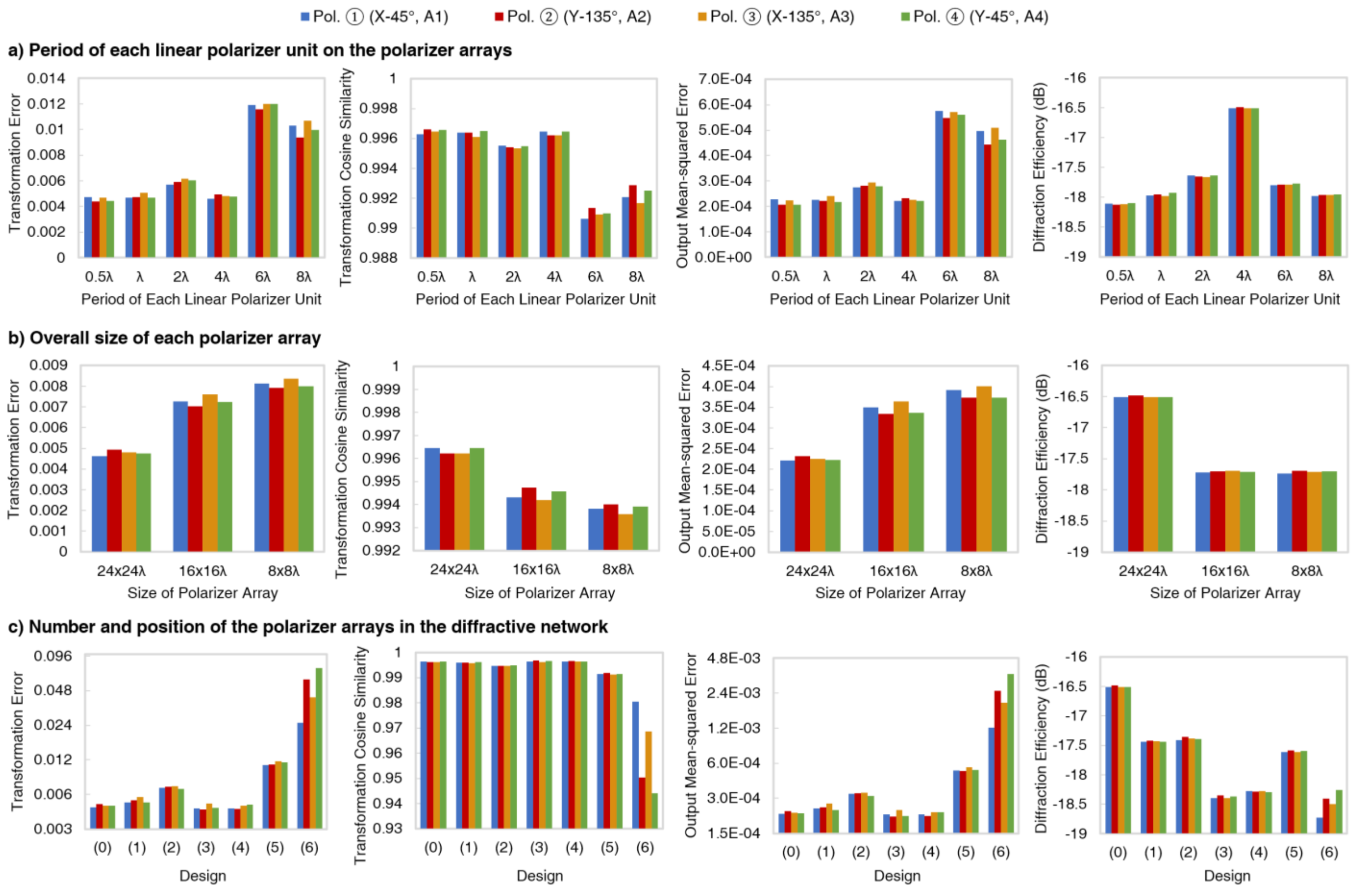

Supplementary Figure SN2.

Here, we analyze the impact of some physical parameters of the polarizer arrays on the computational performance of polarization multiplexed diffractive networks. As our testbed, we used the 4-channel polarization multiplexed system introduced in the main text with $N = N_p N_i N_o = 16.3k$ and the same four target linear transforms (i.e., $\boldsymbol{A_1}$, $\boldsymbol{A_2}$, $\boldsymbol{A_3}$ and $\boldsymbol{A_4}$), i.e., $N_p = 4$. As shown in Supplementary Fig. SN2, we explored three different comparisons to perform this analysis by varying (a) the period of each linear polarizer unit on the polarizer array, (b) the overall size of each polarizer array, and (c) the number and position of these polarizer arrays within the diffractive network. All the parameter tuning used here is performed based on the $N_p = 4$ system that we reported in the Methods section of the main text.

Since the number and position of the polarizer arrays within a diffractive network can be arranged in various combinations, here we selected the following seven designs for comparison, each of which was assigned a unique notation/number in Supplementary Fig. SN2c:

- "(0)" refers to the diffractive model used in the main text ($N_p = 4$), which has 2 polarizer arrays placed after the 3rd and 5th diffractive layers;
- "(1)" has 2 polarizer arrays placed after the 4th and 5th diffractive layers;

- "(2)" has 2 polarizer arrays placed after the 3rd and 6th diffractive layers;
- "(3)" has 4 polarizer arrays placed after the 3rd to 6th diffractive layers;
- "(4)" has 6 polarizer arrays placed after the 2nd to 7th diffractive layers;
- "(5)" has 8 polarizer arrays placed after all the diffractive layers;
- "(6)" has only one polarizer array placed after the 5th diffractive layer.

Based on this comparative analysis reported in Supplementary Fig. SN2, we can summarize our observations as follows:

1. A better all-optical approximation accuracy can be achieved when the period of each linear polarization unit on the polarizer array is $\leq 4\lambda$, and a period of ~4λ empirically appears as an optimal choice, also providing an improved output diffraction efficiency;

2. The transformation accuracy and the diffraction efficiency of the system can be optimized by using polarizer arrays with a sufficiently large size, i.e., at least matching the size/width of the neighboring trainable diffractive layers;

3. Using two polarizer arrays and placing them apart with an axial distance of ~8λ within the diffractive volume can provide improved results for the all-optical transformation accuracy and the diffraction efficiency of $N_p = 4$ designs;

4. Using too many (e.g., >6) or too few (e.g., 1) polarizer arrays will considerably deteriorate the computational accuracy of our diffractive processor for $N_p = 4$.

• Supplementary Note 3: Polarization Extinction Ratio (PER)-related analysis of polarization multiplexed diffractive networks

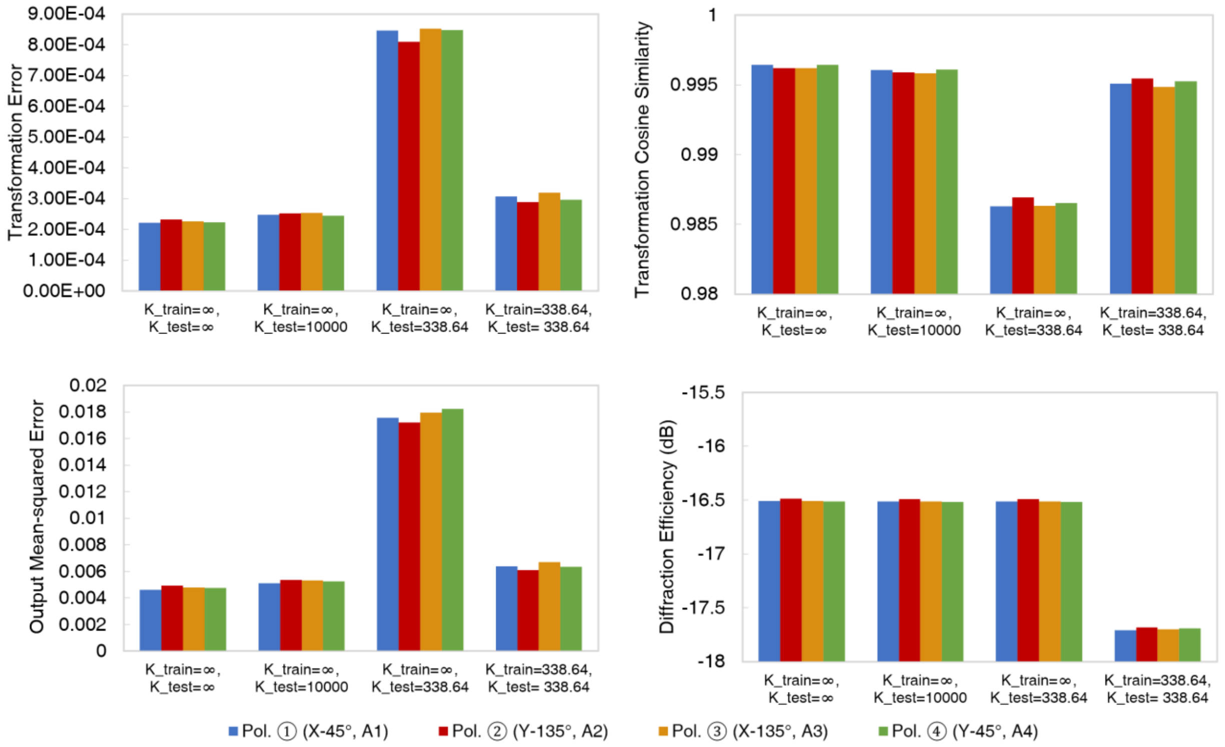

Supplementary Figure SN3.

(1) Here we take the 4-channel polarization multiplexed diffractive processor (depicted in Fig. 6 of the main text) as our testbed to analyze how the modeling of the PER (κ) of the polarizer arrays would affect the computational performance of our diffractive system. We used two different PERs, i.e., κ = 10^5 and κ = 338.64, where these PER values reflect (1) the typical PER of commercial linear polarizers[1] and (2) the PER of a microscopic polarizer filter array used in polarization-based CMOS image sensors[2], respectively. We can model the linear polarizer elements on each polarizer array/seed with a finite PER in the following form[3,4]:

$$J_{\text{linear}}(x,y,z) = \begin{bmatrix} \cos^2\theta(x,y,z) + \kappa^{-1/2}\sin^2\theta(x,y,z) & (1-\kappa^{-1/2})\cos\theta(x,y,z)\,\sin\theta(x,y,z) \\ (1-\kappa^{-1/2})\sin\theta(x,y,z)\,\cos\theta(x,y,z) & \sin^2\theta(x,y,z) + \kappa^{-1/2}\cos^2\theta(x,y,z) \end{bmatrix} \quad (\text{S17}).$$

Using Eq. (S17) as part of our optical forward model, the computational performance of our polarization multiplexed diffractive processors ($N_p = 4$) as a function of different PER values is reported in Supplementary Fig. SN3. It can be seen that even for a small PER value of 338.64, by appropriately training the polarization multiplexed diffractive network using κ_{train} = 338.64, the all-optical transformation accuracy of the diffractive network can approximately match the ideal case of $\kappa_{\text{train}} = \infty$, $\kappa_{\text{test}} = \infty$ (Supplementary Fig. SN3). Furthermore, our results also reveal that a relatively large PER of κ = 10^5 has a negligible impact on the linear transformation performance of the diffractive network compared to the ideal case of ($\kappa_{\text{train}} = \infty$, $\kappa_{\text{test}} = \infty$).

(2) We can also consider each polarization multiplexed diffractive processor as a monolithic polarization optical element and quantify the overall PER of the diffractive network after its training. We should note that for the SeqPA mode of operation, PER is not a meaningful figure-of-merit for a polarization multiplexed diffractive network since only one orthogonal polarization state is read/measured at a given time due to the sequential access of each target transformation through the diffractive network. In other words, the SeqPA mode of operation does not penalize the leakage of power into an orthogonal polarization state at the output field-of-view as it does not impact the accuracy of each all-optical transformation that is sequentially performed. Therefore, this PER analysis for the entire diffractive network treated as a single polarization optical element was only applied to the SimPA-based 2-channel polarization multiplexed diffractive design, which resulted in an overall PER of 51726.596 at the output field-of-view of the diffractive network.

Such a high PER is expected since the SimPA mode is designed to *simultaneously* perform two different linear transformations using two orthogonal polarization states, and therefore undesired polarization cross-talk at the output field-of-view was penalized during the training phase, successfully leading to the observed high PER value.

Supplementary Figures

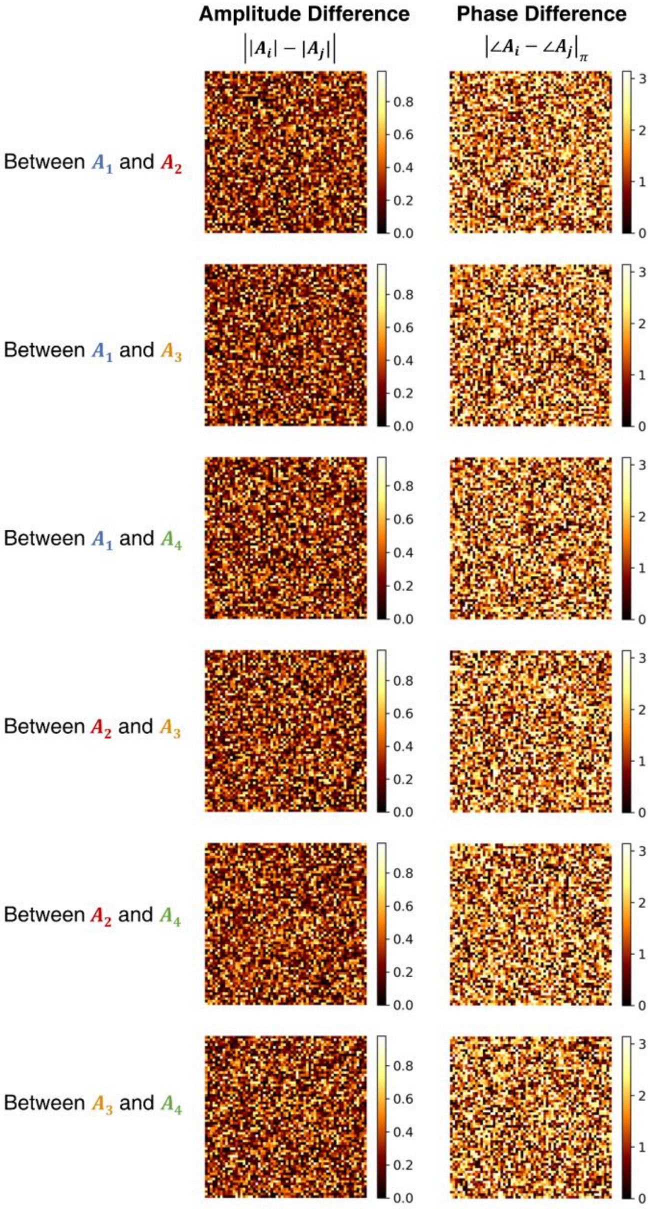

Figure S1. Phase and amplitude differences between the linear transformation matrices A_1, A_2, A_3 and A_4.

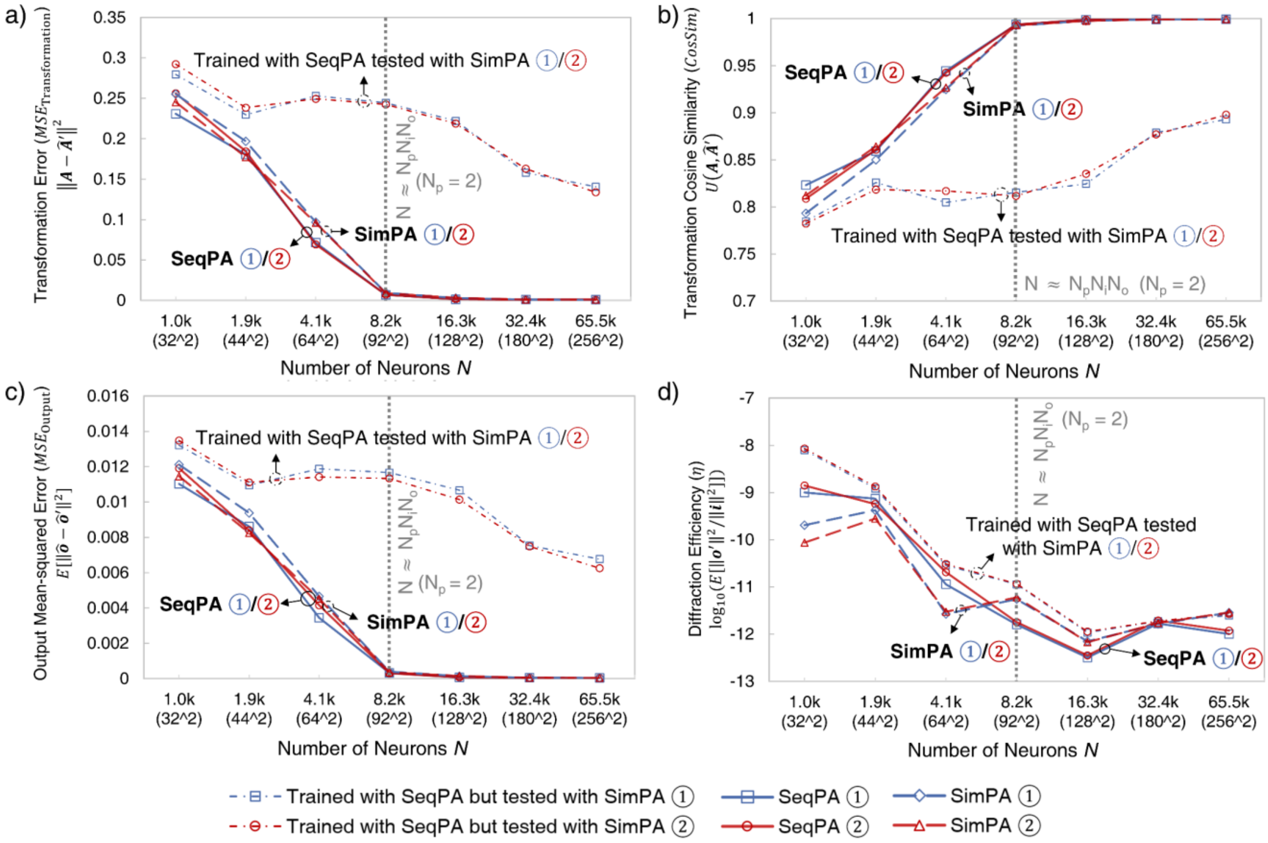

Figure S2. Diffractive all-optical transformation results of blindly testing the SeqPA-trained 2-channel polarization multiplexed diffractive networks under the SimPA mode. a, Curves representing the normalized mean-squared errors between the ground truth transformation matrices ($\boldsymbol{A}_1$ and $\boldsymbol{A}_2$, shown in Figs. 2a and 3) and their all-optical transforms ($\boldsymbol{A}_1'$ and $\boldsymbol{A}_2'$) resulting from the trained diffractive networks as a function of N. The dash-dotted curves (labeled with "Trained with SeqPA but tested with SimPA ①/②") are achieved by the 2-channel polarization multiplexed diffractive network trained under the SeqPA mode but then immediately tested under the SimPA mode, which are compared with the solid curves achieved by the same diffractive networks both trained and tested under the SeqPA mode (labeled with "SeqPA ①/②"), and the long dashed curves achieved by different diffractive networks both trained and tested under the SimPA mode (labeled with "SimPA ①/②"). Note that the results for the two polarization channels ① and ②, corresponding to transforms $\boldsymbol{A}_1'$ and $\boldsymbol{A}_2'$, are shown in separate curves but jointly labeled with "… ①/②", respectively, due to the spatial overlap of these curves. The space between the simulation data points is linearly interpolated. **b**, Same as (b), but the cosine similarity between the all-optical transforms and their ground truth shown in (a) is reported. **c**, Same as (b), but the mean-squared error between the diffractive network-estimated output fields and their ground truth is reported. **d**, Diffraction efficiency of the presented diffractive networks

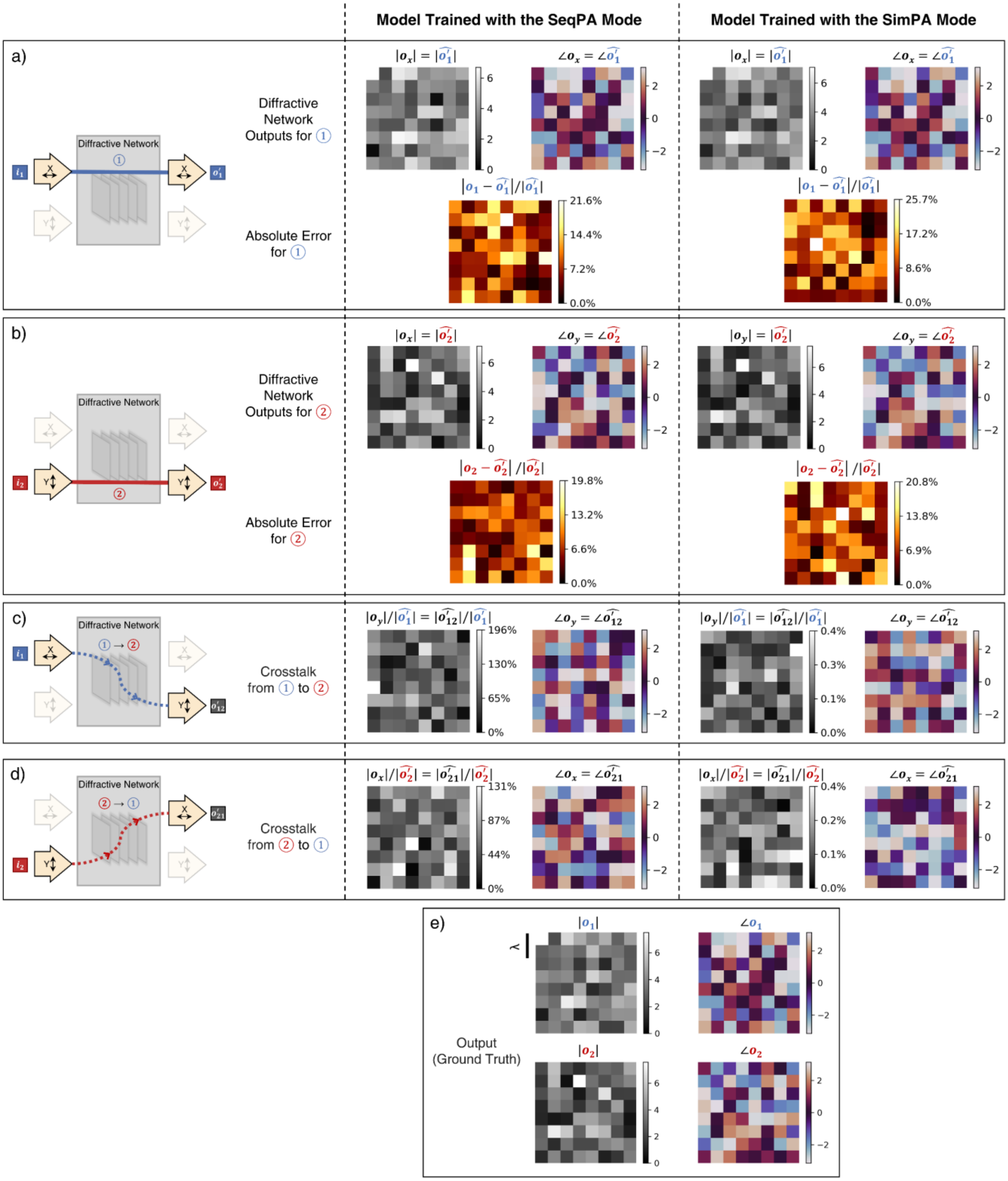

Figure S3. Crosstalk analysis of the 2-channel polarization multiplexed diffractive networks trained using the SeqPA and SimPA modes. Note that $\boldsymbol{o}'_{12}$ represents the crosstalk component from the polarization channel ① to ② measured at the output field of the diffractive network, while $\boldsymbol{o}'_{21}$ represents the crosstalk component from the polarization channel ② to ① measured at the output field of the diffractive network.

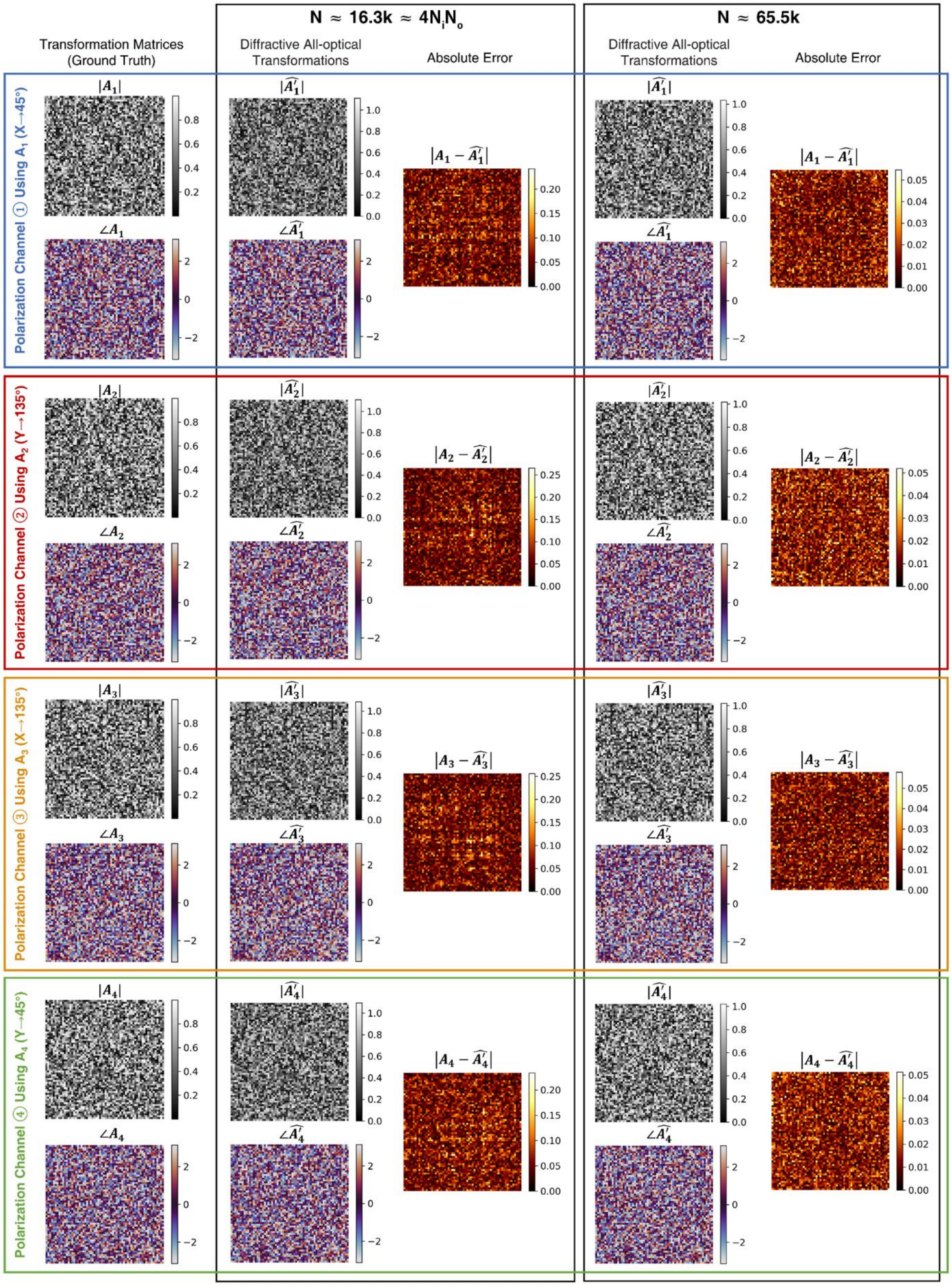

Figure S4. All-optical transformation matrices estimated by the 4-channel polarization multiplexed diffractive designs with *N* = 16.3k and 65.5k, and their differences from the ground truth matrices.

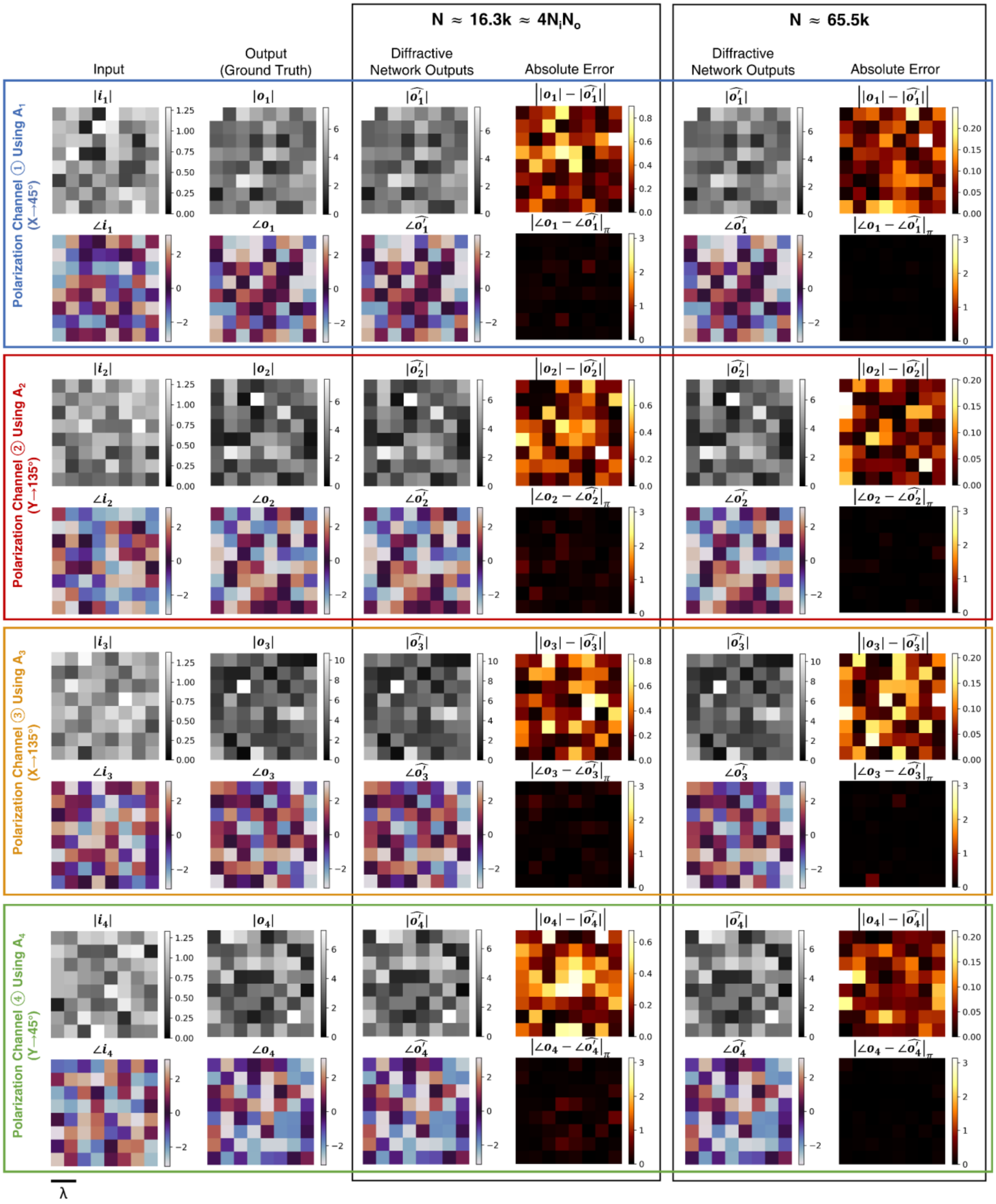

Figure S5. Examples of input/output complex fields for the ground truth transformations presented in Figs. 7 and S4 along with the output fields computed by the 4-channel polarization multiplexed diffractive designs using $N \approx$ 16.3k and 65.5k. Note that $\left|\angle \boldsymbol{o} - \angle \widehat{\boldsymbol{o}'}\right|_{\pi}$ indicates the wrapped phase difference between the ground truth output field $\boldsymbol{o}$ and the normalized diffractive network output field $\widehat{\boldsymbol{o}'}$

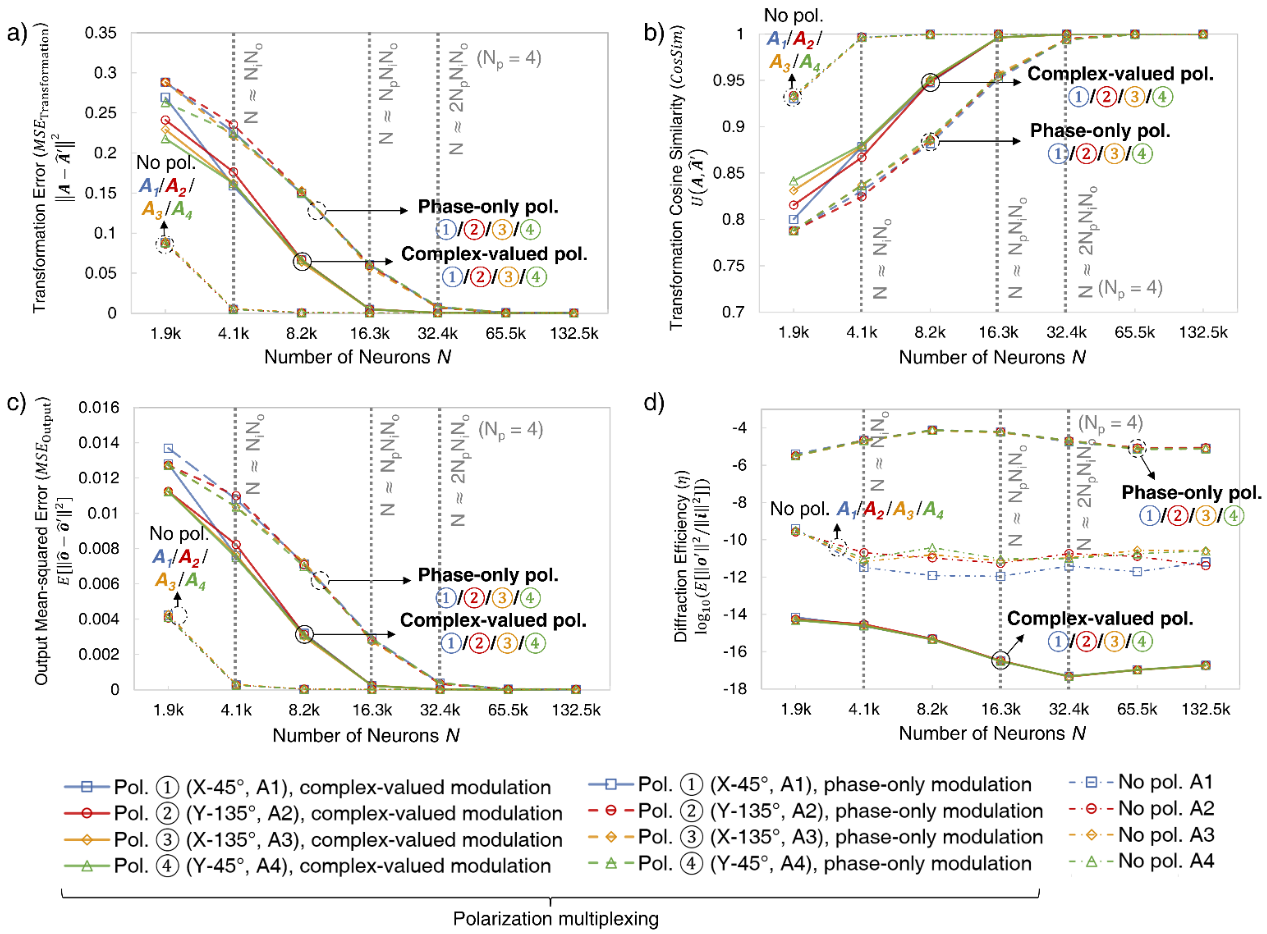

Figure S6. Diffractive all-optical transformation results for 4-channel polarization multiplexing of four distinct arbitrary linear transforms (depicted in Fig. 6) using phase-only diffractive networks. a, Curves representing the normalized mean-squared error between the ground truth transformation matrices ($\boldsymbol{A}_1$, $\boldsymbol{A}_2$, $\boldsymbol{A}_3$ and $\boldsymbol{A}_4$) and the all-optical transforms ($\boldsymbol{A}_1'$, $\boldsymbol{A}_2'$, $\boldsymbol{A}_3'$ and $\boldsymbol{A}_4'$) resulting from the trained diffractive networks as a function of N. The solid curves are achieved by the 4-channel polarization multiplexed, phase-only diffractive networks, which are compared with the dashed curves achieved by the 4-channel polarization multiplexed, complex-valued diffractive networks, and the dash-dotted curves achieved by the regular diffractive networks (without polarization multiplexing). For the polarization multiplexed phase-only diffractive models, the results for the four polarization channels ①, ②, ③ and ④ are shown in separate curves but jointly labeled with "Phase-only pol. ①/②/③/④" due to the spatial overlap of these curves. For the polarization multiplexed complex-valued diffractive models, the results for the four polarization channels ①, ②, ③ and ④ are shown in separate curves but jointly labeled with "Complex-valued pol. ①/②/③/④" due to the spatial overlap of these curves. For the regular diffractive models without polarization multiplexing, the results for all-optical approximation of $\boldsymbol{A}_1$, $\boldsymbol{A}_2$, $\boldsymbol{A}_3$ and $\boldsymbol{A}_4$ (individually) are shown in separate curves but jointly labeled with "No pol. $\boldsymbol{A}_1/\boldsymbol{A}_2/\boldsymbol{A}_3/\boldsymbol{A}_4$" due to the spatial overlap of these curves. The space between the simulation data points is linearly interpolated. **b**, Same as (a) but cosine similarity between the all-optical transforms and their ground truth is reported. **c**, Same as (a) but the mean-squared error between the diffractive network output fields and their ground truth is reported. **d**, Diffraction efficiency of the presented diffractive networks.

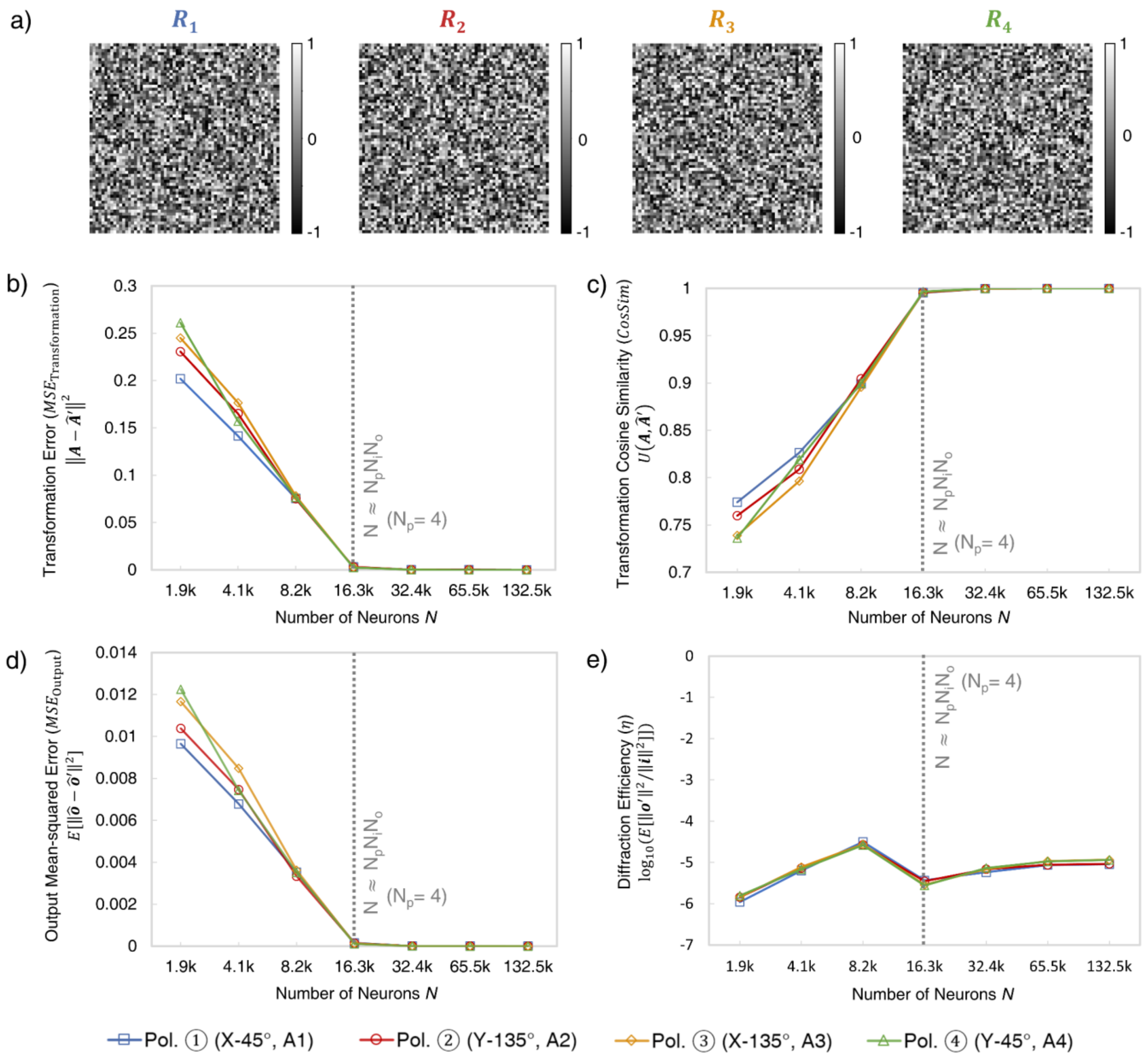

Figure S7. Diffractive all-optical transformation results for 4-channel polarization multiplexing of four distinct arbitrarily-selected real-valued linear transforms using phase-only diffractive networks. a, Arbitrarily-selected real-valued matrices $\boldsymbol{R}_1$, $\boldsymbol{R}_2$, $\boldsymbol{R}_3$ and $\boldsymbol{R}_4$, which serve as the ground truth (target) for the diffractive all-optical transformations. **b**, Curves representing the normalized mean-squared error between the ground truth transformation matrices ($\boldsymbol{R}_1$, $\boldsymbol{R}_2$, $\boldsymbol{R}_3$ and $\boldsymbol{R}_4$) and the all-optical transforms ($\boldsymbol{R}'_1$, $\boldsymbol{R}'_2$, $\boldsymbol{R}'_3$ and $\boldsymbol{R}'_4$) resulting from the trained phase-only diffractive networks as a function of N. The space between the simulation data points is linearly interpolated. **c**, Same as (b) but cosine similarity between the all-optical transforms and their ground truth is reported. **d**, Same as (b) but the mean-squared error between the diffractive network output fields and their ground truth is reported. **e**, Diffraction efficiency of the presented diffractive networks.

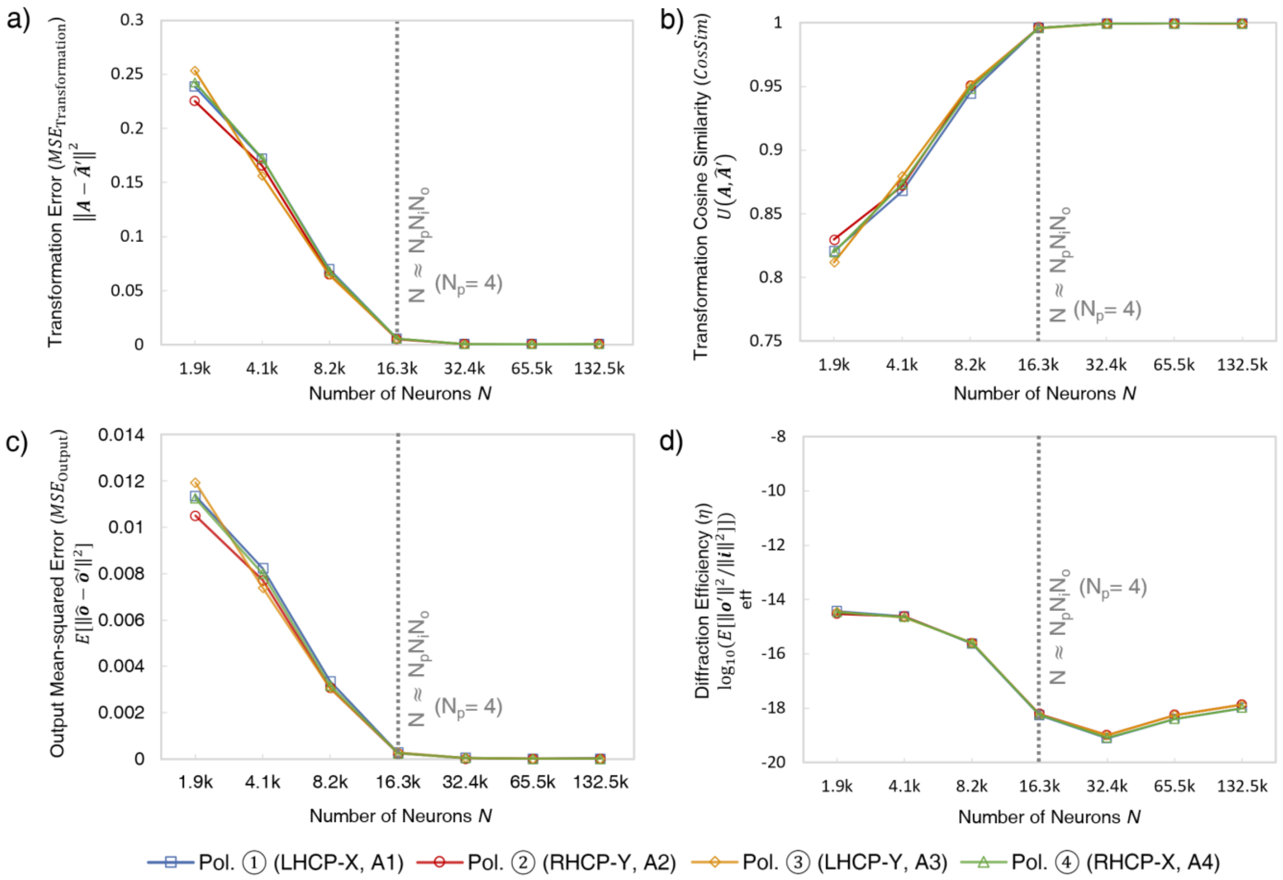

Figure S8. Diffractive all-optical transformation results for 4-channel polarization multiplexing of four distinct arbitrary linear transforms using orthogonal circular polarization states at the input field-of-view. Left- and right-hand circular polarization states (i.e., LHCP and RHCP) are used at the input of a polarization-multiplexed diffractive network to encode the input information, and x and y linear polarization states are used at the output of the diffractive network. 4 different, arbitrarily-selected, complex-valued linear transformations were each assigned to one combination of circular-linear polarization. **a**, Curves representing the normalized mean-squared error between the ground truth transformation matrices ($\boldsymbol{A_1}$, $\boldsymbol{A_2}$, $\boldsymbol{A_3}$ and $\boldsymbol{A_4}$, shown in Fig. 7a of the main text) and the all-optical transforms ($\boldsymbol{A}'_1$, $\boldsymbol{A}'_2$, $\boldsymbol{A}'_3$and $\boldsymbol{A}'_4$) resulting from the trained diffractive networks as a function of N. The space between the simulation data points is linearly interpolated. **b**, Same as (b) but the cosine similarity between the all-optical transforms and their ground truth is reported. **c**, Same as (b) but the mean-squared error between the diffractive network output fields and their ground truth is reported. **d**, Diffraction efficiency of the presented diffractive networks.

References

1 High Performance Glass Linear Polarizers | Edmund Optics. https://www.edmundoptics.com/f/high-contrast-glass-linear-polarizers/12725/ (accessed 19 Apr2022).

2 Phoenix 5.0MP Polarsens Polarization Camera, Sony's IMX250MZR and IMX250MYR CMOS | LUCID Vision Labs. https://thinklucid.com/product/phoenix-5-0-mp-polarized-model/ (accessed 19 Apr2022).

3 Brecha RJ, Pedrotti LM. Analysis of imperfect polarizer effects in magnetic rotation spectroscopy. *Opt Express* 1999; **5**: 101–113.

4 Huang C, Zhao S, Chen H, Liao Z. Attenuation characterization of multiple combinations of imperfect polarizers. *JOSA A* 2010; **27**: 1060–1068.
